# Development of novel broad-spectrum antimicrobial lipopeptides derived from plantaricin NC8 β

**DOI:** 10.1038/s41598-023-31185-8

**Published:** 2023-03-13

**Authors:** Emanuel Wiman, Elisa Zattarin, Daniel Aili, Torbjörn Bengtsson, Robert Selegård, Hazem Khalaf

**Affiliations:** 1 School of Medical Sciences, Faculty of Medicine and Health, Department of Microbiology, Immunology and Reproductive Science, Örebro University, Örebro, Sweden; 2 Laboratory of Molecular Materials, Division of Biophysics and Bioengineering, Department of Physics, Chemistry and Biology, Linköping University, 581 83 Linköping, Sweden

**Keywords:** Antimicrobials, Peptides, Microbiology, Pathogens

## Abstract

Bacterial resistance towards antibiotics is a major global health issue. Very few novel antimicrobial agents and therapies have been made available for clinical use during the past decades, despite an increasing need. Antimicrobial peptides have been intensely studied, many of which have shown great promise in vitro. We have previously demonstrated that the bacteriocin Plantaricin NC8 αβ (PLNC8 αβ) from *Lactobacillus plantarum* effectively inhibits *Staphylococcus* spp., and shows little to no cytotoxicity towards human keratinocytes. However, due to its limitations in inhibiting gram-negative species, the aim of the present study was to identify novel antimicrobial peptidomimetic compounds with an enhanced spectrum of activity, derived from the β peptide of PLNC8 αβ. We have rationally designed and synthesized a small library of lipopeptides with significantly improved antimicrobial activity towards both gram-positive and gram-negative bacteria, including the ESKAPE pathogens. The lipopeptides consist of 16 amino acids with a terminal fatty acid chain and assemble into micelles that effectively inhibit and kill bacteria by permeabilizing their cell membranes. They demonstrate low hemolytic activity and liposome model systems further confirm selectivity for bacterial lipid membranes. The combination of lipopeptides with different antibiotics enhanced the effects in a synergistic or additive manner. Our data suggest that the novel lipopeptides are promising as future antimicrobial agents, however additional experiments using relevant animal models are necessary to further validate their in vivo efficacy.

## Introduction

Antibiotics are the most effective treatment against bacterial infections of both gram-positive and gram-negative species. Many species are opportunistic pathogens that may cause severe infections in humans in connection with chronic wounds and medical devices, e.g., catheters and prosthetic implants^1^. These bacterial accumulations are the basis of persistent infections that are generally difficult to treat, which increases the risk for bacterial dissemination and development of systemic complications^2,3^. Furthermore, considering the gradual increase in antibiotic resistance, treatment may be even more difficult to achieve as the available options are becoming increasingly limited^4^. Consequently, new approaches and innovative alternative treatments against bacterial infections are urgently needed. Antimicrobial peptides (AMPs) represent one of the most promising classes of antimicrobial substances and a rich source for development of highly potent new therapeutics^5,6^.

Since antibiotics are becoming less effective, AMPs have become attractive candidates in human medicine due to their antimicrobial properties. Many AMPs display low toxicity towards eukaryotic cells, and activity against pathogenic bacteria that have acquired resistance to antibiotics^5,7^. These peptides generally consist of short sequences (< 100 amino acids) with no ordered secondary structure when in solution. They are typically very heat stable and tolerate changes in pH, and express bactericidal activity against a wide range of microbes^8–11^. Bacteriocins are a heterogenous group of AMPs produced by several different bacteria. They can be both narrow- and broad-spectrum, and several bacteriocins exhibit potent antibacterial activity, and low toxicity to animal cells^5^. We have previously shown that the bacteriocin PLNC8 αβ permeabilizes the gram-negative oral pathogen *Porphyromonas gingivalis* and inhibits its cytotoxic and immunomodulatory effects on human cells^12,13^. Furthermore, we have recently shown that PLNC8 αβ is most effective against bacteria of the genus *Staphylococcus*, including strains that have acquired resistance to antibiotics, and enhances severalfold the activity of different antibiotics^14^.

The most problematic and disease-causing bacteria have been categorized by the World Health Organization (WHO) and constitute *Enterococcus faecium, Staphylococcus aureus, Klebsiella pneumoniae, Acinetobacter baumannii, Pseudomonas aeruginosa,* and *Enterobacter* species (ESKAPE). These bacteria have developed multidrug resistance against several antibiotic classes and can form biofilms. WHO has listed the ESKAPE pathogens among its top 12 priority pathogens in urgent need for the development of new antimicrobials. Most of the antibiotics recommended in the Clinical & Laboratory Standards Institute guidelines to act against the ESKAPE pathogens have been removed from the guidelines, and very few novel antibiotics or antibiotic combinations have been added in their place^15^. Since infections caused by the ESKAPE pathogens are the most problematic infections in humans, it is important to find alternative treatments consisting of novel antimicrobial compounds. The potency and low toxicity of new and innovative substances may potentially reduce the overall use of antibiotics, and consequently the development and spreading of antimicrobial resistance may be suppressed by using AMPs, either alone or in combination with low doses of antibiotics.

AMPs have been intensely studied for decades. While numerous compounds have been shown to be highly effective in vitro, the majority have shown limitations such as susceptibility to proteases, and the inhibition of activity when in the presence of salts or cations or subjected to changes in pH, when examined in more complex milieus^16,17^. However, advancements in the field of drug engineering and antimicrobial peptidomimetics now allow for the design of AMP-mimetics with improved protease stability, lower toxicity, and higher antimicrobial activity, thus providing means to circumvent these limitations^18,19^.

In light of this, we have designed and carried out a careful and systematic optimization and characterization of a novel class of peptidomimetic compounds derived from Plantaricin NC8β, with the aim to identify novel antimicrobial compounds with low toxicity and improved antimicrobial activity towards a broader spectrum of bacterial species, with a focus on future topical applications. We show that several of these novel compounds, comprised of 16 amino acids with a short terminal fatty acid (FA) chain, exhibit significant antibacterial activity and low hemolytic activity to human erythrocytes. The lipopeptides demonstrate higher affinity for bacterial membrane-mimicking lipid bilayers as compared to mammalian-mimicking cell membranes, making them interesting candidates for further research. Furthermore, we suggest that the systematic design approach developed in this study allow for development of novel antimicrobial compounds, where several of the inherent limitations of antimicrobial peptides can be circumvented.

## Material and methods

### Bacterial culture

*S. aureus* (ATCC 29213, MSSA, ATCC, Manassas, VA), *E. coli* (K-12 MG1655) and clinical isolates of *S. aureus, E. coli*, *E*. *faecium*, *K. pneumoniae*, *A. baumannii*, *P. aeruginosa* and *E. cloacae* obtained from the Department of Laboratory Medicine at Örebro University Hospital were streaked on Luria–Bertani (LB) agar plates and incubated at 37 °C overnight. Single colonies were inoculated into 5 ml of LB broth and incubated on a shaker (400 rpm) at 37 °C overnight. The bacterial concentrations were determined by viable count and were adjusted to correlate with approximately 10^9^ CFU/ml. Resistance patterns for the clinical isolates can be found in Supplementary Table S1.

### Peptide design and prediction of antimicrobial activity

The sequence of PLNC8 β of the two peptide bacteriocin PLNC8 αβ was used to design new and optimized antimicrobial peptides. The antimicrobial activity of full length and truncated peptides of PLNC8 β was predicted using the servers AntiBP^20^ and ADAM^21^. The sequence with the highest score of predicted antimicrobial activity was used to generate new variants. Peptide characteristics, such as iso-electric point, net charge at pH 7, and percentage of hydrophobic, acidic, basic, and neutral residues were determined using Peptide 2.0 (Custom Peptide Synthesis (peptide2.com)). A theoretical assumption of peptide structure was predicted using the PEP-FOLD tool^22,23^ in the RPBS Web Portal^24^. All the sequences showing characteristics of being antimicrobial peptides were synthesized and tested against gram-positive (*S. aureus*) and gram-negative (*E. coli*) bacteria.

### Peptide and lipopeptide synthesis

All peptides and lipopeptides were synthesized using Fmoc-chemistry on an automated microwave peptide synthesizer (Liberty Blue, CEM) in 25–250 μM scale. ProTide Rinkamide (LL) resin was used as solid support for all synthesis yielding an amidated C-terminal. The peptides LL-37 and PLNC8 β with a free acid C-terminal was synthesized on a Cl-MPA ProTide (LL) resin using anhydrous KI (0.125 M) and DIEA (1 M) in DMF to attach the first Fmoc-protected amino acid (5 eq). The reaction was performed twice for 10 min at 90 °C using microwave conditions. For all peptides, Fmoc-protected amino acids were sequentially coupled using a five-fold excess of amino acid, Oxyma as base and DIC as coupling reagent in DMF under microwave conditions. Fmoc-deprotection was achieved by treatment with 20% Piperidine in DMF under microwave conditions. After final Fmoc-deprotection N-terminal modification was achieved by treatment of the resin-bound peptide with acetic anhydride in DMF (1:1) for acetylation, or n-alkane acids (10 eq) combined with HCTU (10 eq) and DIEA (20 eq) in DMF for lipidation. Global deprotection and cleavage of peptides from resin was achieved by treatment with TFA:TIS:H_2_O (95/2.5/2.5, v/v/v) for 3 h before being concentrated using a stream of nitrogen. The crude peptides were precipitated in ice cold diethyl ether, twice, and the ether was discarded. The crude peptides were purified on a semi preparative HPLC system (Dionex) equipped with a RP C-18 column (ReproSil Gold) using an aqueous gradient of acetonitrile containing 0.1% TFA. Purity was controlled using an analytical column (C-18, Supelcosil) attached to the same HPLC system (Supplementary Fig. S1) and peptide identity was confirmed using Maldi-ToF mass spectrometer (Bruker) (Supplementary Fig. S2).

### Liposome preparation

Liposomes were prepared by thin-film hydration method. Lipids stock solutions (10 mg/ml in chloroform, Avanti Polar Lipids, Inc., Alabaster, United States) of the lipids 1-palmitoyl-2-oleoyl-sn-glycero-3-phosphocholine (POPC), 1-palmitoyl-2-oleoyl-sn-glycero-3-phospho-L-serine (POPS), 1-Hexadecanoyl-2-(9Z-Octadecenoyl)-sn-Glycero-3-Phosphoglycerol (POPG) and Cholesterol (Chol) were employed. Bacterial liposomes were prepared with a 75:25 ratio of POPC:POPG and mammalian liposomes were prepared with a 65:5:30 ratio of POPC:POPS:Chol. Lipid solutions were thoroughly homogenised, and the solvent was evaporated with the use of a dry N_2_ steam to yield a lipid cake. Complete drying was achieved with an overnight incubation in a vacuum desiccator. The lipid film was subsequently hydrated with a 5(6)-CF solution (50 mM CF, 10 mM PBS, 90 mM NaCl, pH 7.4). Following a 10 min incubation on orbital shaker (50 min^−1^), the solution was vortexed for 60 s on medium speed. A mini extruder (Avanti Polar Lipids, Inc., United States) was employed and 21 extrusions were performed through a 0.1 µm filter membrane (Nuclepore track-etched hydrophilic membrane, Cytiva, Whatman, MA, United States), resulting in monodispersed unilamellar liposomes. Directly before use the liposomes were purified from unencapsulated CF by filtration through a gel filtration column (PD Minitrap G-25 column, Cytiva, United States), against PBS buffer (10 mM, pH 7.4).

### Carboxyfluorescein release assay

Peptide and lipopeptide activity towards liposome models were evaluated using a carboxyfluorescein (CF) release assay. Liposomes were diluted in PBS (10 mM, pH 7.4) to a final lipid concentration of 25 µM and incubated with peptides (10^–5^–10^2^ µM) in a 96-well plate (n = 3). CF release was monitored using an Infinite M1000 PRO plate reader every 2.5 min for 1 h. Full (100%) CF release was obtained by addition of 1% Triton X-100 solution followed by 10 min incubation. CF release percentage was evaluated according to the formula 100 * (F − F_0_)/(F_T_ − F_0_), where F indicates the instantaneous fluorescence, F_T_ the total fluorescence and F_0_ the background fluorescence prior to peptide addition. Data were fitted to a Hill-1 curve using MATLAB R2019a (The MathWorks Inc., Natick, Massachusetts, United States) and the half maximum effective concentration (EC_50_) was extracted.

### Circular dichroism spectroscopy

Circular dichroism (CD) spectroscopy was utilized to determine the structure of peptides upon interaction with lipid membranes. A Chirascan (Applied Photophysics, Leatherhead, United Kingdom) was utilized, and measurements were conducted using a 1 mm light path quartz cuvette at ambient temperature, in the wavelength range 195–280 nm (0.5 nm steps). Bacterial membrane-mimicking liposomes (POPC:POPG 75:25) were prepared in PBS buffer (10 mM, pH 7.4) as previously described. Lipopeptides *L*-6-C5 and *L*-6-C5-Leu were mixed with liposome solution and incubated for 30 min. In all experiments, the peptide concentration was 30 µM and the lipid concentration was 1.2 mM. Three scans were recorded for each sample and results were baseline corrected against PBS buffer (10 mM, pH 7.4). Curves were analysed with MATLAB R2019a (The MathWorks, Inc., Natick, Massachusetts, United States) and smoothed with a Savitzky-Golay filter.

### Dynamic light scattering (DLS)

Lipopeptide aggregation was evaluated on a ALV/CGS-8F Platform (ALV-GmbH, Langen, Germany) equipped with a 632.8 mm He–Ne laser. Scattered light was collected at 90°. PBS (10 mM, pH 7.4) was chosen as buffer, and filtered with a 0.22 µm filter prior to use. The samples were prepared in a cylindrical glass cuvette immersed in refractive-index matching toluene. The peptides were serially diluted between 100 µM and 0.01 nM, sonicated for 1 min and incubated for 5 min at 22 °C and vortexed prior to measuring. The temperature was controlled throughout the experiment with the use of a circulating water bath. Data were analysed with ALV-Correlator (Version 3.0, ALV-GmbH, Langen, Germany) software, and the scattering intensity was obtained through an average of 10 consecutive 30 s runs.

### Antimicrobial activity

The broth microdilution method was used to determine minimal inhibitory concentration (MIC) and minimal bactericidal concentration (MBC), in accordance with the EUCAST standards for the broth microdilution method. Two-fold serial dilutions of the lipopeptides were used, and the final concentrations ranged from 0.19 to 100 µM. In brief, two-fold serial dilutions of the lipopeptides were performed in a 96-well plate in PBS, with a final volume of 100 µl per well, after which 100 µl of LB-broth media containing approximately 5 × 10^5^ CFU/ml of bacteria was added to each well. The plate was then incubated for 20 h in 37 °C on a shaker (400 rpm). The effect of lipopeptide-antibiotic combinations was investigated by performing checkerboard assays. The assays were performed in accordance with the MIC and MBC tests conducted above, but with horizontal and longitudinal two-fold serial dilutions of the tested compounds. The final concentrations of the antibiotics vancomycin, tetracycline, ciprofloxacin, and rifampicin ranged between 0.031 and 8 µg/ml (*S. aureus*), while for *E. coli*, gentamicin, cefotaxime and ciprofloxacin ranged between 0.0078 and 4 µg/ml and rifampicin ranged between 0.78 and 100 µg/ml. All MIC-values were determined visually and spectroscopically (620 nm) as the first concentration that completely inhibited bacterial growth. All MBC values were determined by culturing 10 µl drops of all concentrations that resulted in complete inhibition of bacterial growth on LB-agar plates, and the lowest concentration where no growth was observed represented the MBC. The fractional inhibitory concentration (FIC) and fractional bactericidal concentration (FBC) were calculated by the equation (MIC or MBC of peptide in combination/MIC or MBC of peptide alone) + (MIC or MBC of antibiotic in combination/MIC or MBC of antibiotic alone). Synergy was defined as FIC/FBC ≤ 0.5, additive when 0.5 < FIC/FBC ≤ 1, indifferent when 1 < FIC/FBC < 2 and antagonistic when FIC/FBC ≥ 2. All experiments were repeated at least three times.

### Resistance development

In order to evaluate the risk of the development of resistance towards the lipopeptide, a serial passage assay was conducted. In brief, *S. aureus* and *E. coli* were cultured in the presence of sub-MIC concentrations (1 µM) of *L*-6-C5 for 30 passages. MIC and MBC were determined for passages 0, 10, 20 and 30, using the broth microdilution method and compared with the unexposed bacteria (passage 0).

### Hemolytic activity

The hemolytic activity of the lipopeptides was investigated by collecting blood from healthy volunteers in heparinized vacutainers. Briefly, the blood was centrifuged at 600 × g for 5 min and the erythrocyte pellet was washed three times in PBS. The cells were then suspended in PBS and added to 96-well plates (15% erythrocyte suspension/well), containing the lipopeptides with two-fold serial dilution. The plates were incubated for 1 h at 37 °C followed by centrifugation for 5 min at 900×*g* and absorbance measurement of the supernatants at 540 nm. Hemolytic activity (%) was calculated by subtracting the negative control from all values and normalization against the positive control (0.5% Triton X-100), that was set to 100%. All experiments, each in duplicate, were repeated three times.

### Microscopy

The fluorescent dye Sytox® Green was used to investigate membrane permeabilization caused by the lipopeptides. This fluorophore can only cross damaged membranes and fluoresce upon binding to nucleic acids. The bacteria were washed and resuspended in PBS and incubated for 5 min with or without peptides in 96-well microtiter plates. Images were captured with Olympus BX41.

### Ethics statement

Ethical permission for collecting heparinized blood from healthy volunteers was approved by the regional ethical board at Örebro-Uppsala County (Dnr 2015/543). Informed consent was obtained from all volunteers. Collection of blood and associated methods were carried out in accordance with relevant guidelines and regulations.

## Results

**Figure 1:**
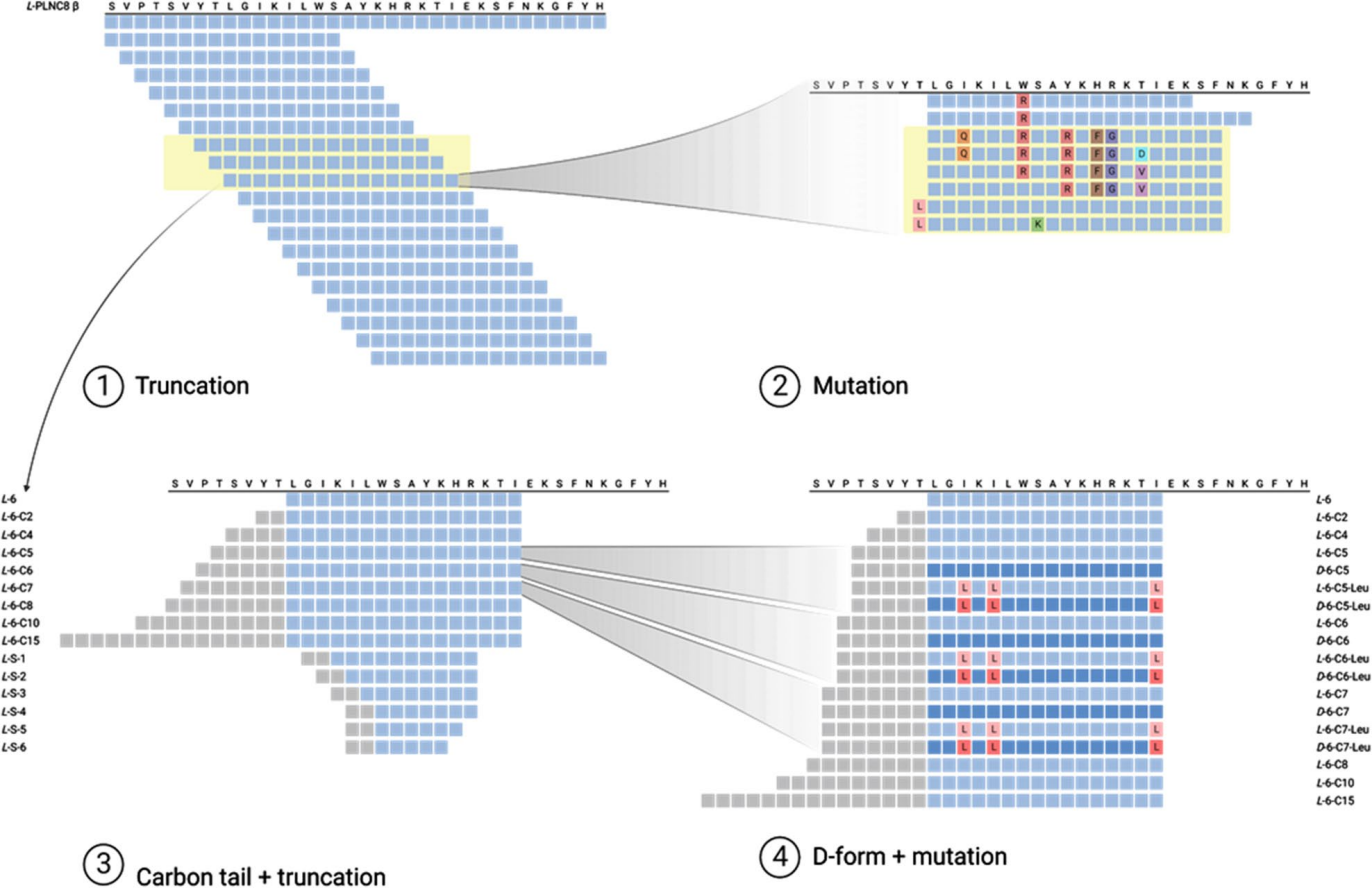
Overview of the design strategy of the lipopeptides. Schematics of the truncation and mutation process of peptide PLNC8 β (sequence written on top). Light green box indicates *L*-form amino acids, dark green box indicates *D*-form amino acids, and grey box indicate carbon atoms. The box highlighted in yellow indicates high predicted antimicrobial activity.

While AMPs are heterogenous in residue length, most natural occurring cationic AMPs typically consists of 12–100 amino acids^25^. Several synthetic cationic AMPs have however been shown to be efficient at residue numbers < 10, which can help solving part of the inherent limitations of several AMPs, namely their bulky size and high synthesis cost^26^. Lata and colleagues^20^, the developers of the AntiBP server, used for part of our predictions, concluded that peptides composed of 15 amino acids was optimal when using their server. An overview of the process described below is shown in Fig. 1. When taking the above points into consideration, with the aim to design as short and effective AMPs as possible, the sequence of PLNC8 β was truncated to 16 amino acids (residue #1–16), after which one amino acid was removed from the N-terminal region and one amino acid added to the C-terminal region. This was completed throughout the entire sequence of PLNC8 β, which is composed of 34 amino acids, and a small library of 19 different peptide sequences was generated, each consisting of 16 amino acids. These sequences where then analyzed in the AntiBP^20^ and ADAM servers^21^ for prediction of antimicrobial activity based on amino acid composition/pattern and sequence-to-structure relationship, respectively. Ten of the sequences were predicted to be antimicrobial peptides. Furthermore, the peptide with the overall highest scoring of the truncated sequences was chosen for residue substitution and similarly evaluated for predicted antimicrobial activity, generating a library of 24 sequences with predicted antimicrobial activity (Table 1).

**Table 1:**
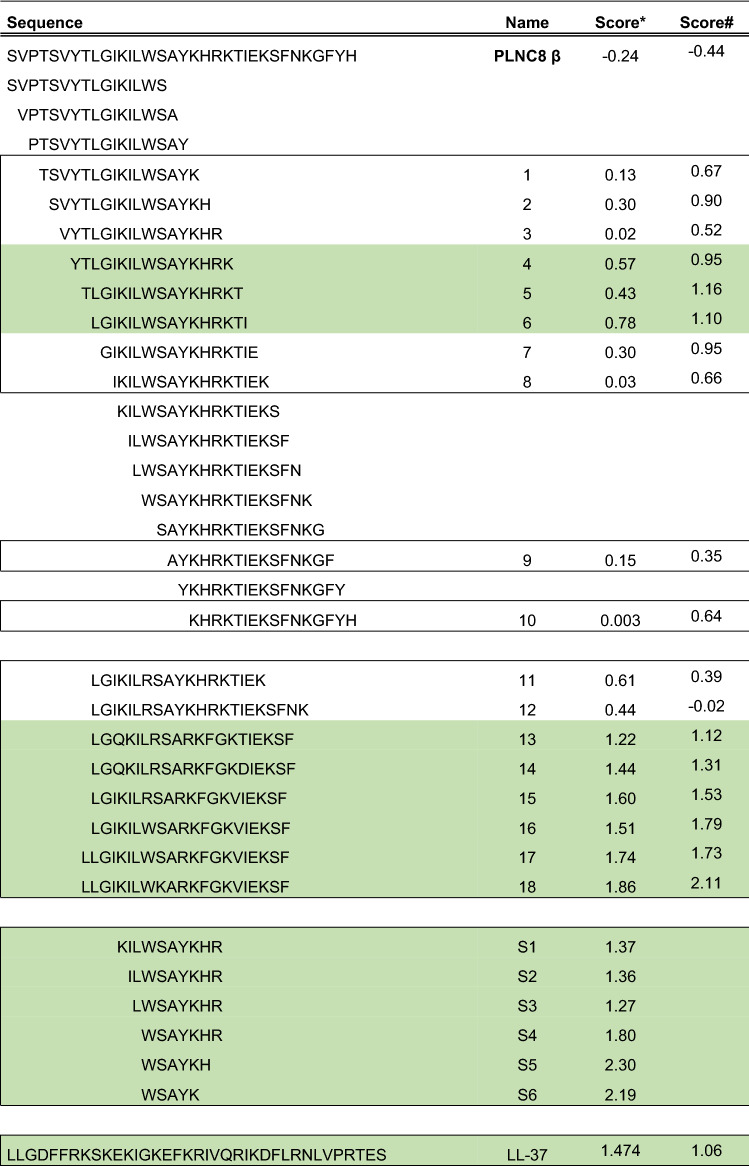
Prediction of the antimicrobial activities of truncated and modified peptides of PLNC8 β.

The antimicrobial activity of full length and truncated peptides of PLNC8 β was predicted using the servers AntiBP*^20^ and ADAM^#^^21^. Peptides that were predicted to have antimicrobial activity are highlighted in boxes and the variants with the highest score, highlighted in green, were synthesized for analysis in biological systems.

The 15 highest scoring peptide sequences from this library were synthesized with an acetylated N-terminal and amidated C-terminal. The antimicrobial activities of these 15 peptides were explored against *S. aureus* and *E. coli*. While all 15 sequences were predicted to have antimicrobial activity, only peptide **6** displayed actual inhibitory and bactericidal activity in vitro against both bacteria with a MIC and MBC of 100 µM for *S. aureus* and 50 µM for *E. coli* (Table 2). The antimicrobial activity of peptide **6** against *E. coli* was comparable to the activity of LL-37, while full-length PLNC8 β showed no activity. Peptide **18** showed bacteriostatic activity against *E. coli* at a final concentration of 100 µM, without displaying any bactericidal activity.

**Table 2:** Antimicrobial activity of full length and truncated peptides of PLNC8 β.

Sequence	Name	MW	*S. aureus*		*E. coli*	
			MIC	MBC	MIC	MBC
H_2_N-SVPTSVYTLGIKILWSAYKHRKTIEKSFNKGFYH	PLNC8 β	4000.6	> 100	> 100	> 100	> 100
CH_3_-CONH-YTLGIKILWSAYKHRK	4	1977,63	> 100	> 100	> 100	> 100
CH_3_-CONH-TLGIKILWSAYKHRKT	5	1915,56	> 100	> 100	> 100	> 100
CH_3_-CONH-LGIKILWSAYKHRKTI	6	1927,62	100	100	50	50
CH_3_-CONH-LGQKILRSARKFGKTIEKSF	13	2308,07	> 100	> 100	> 100	> 100
CH_3_-CONH-LGQKILRSARKFGKDIEKSF	14	2322,05	> 100	> 100	> 100	> 100
CH_3_-CONH-LGIKILRSARKFGKVIEKSF	15	2291,13	> 100	> 100	> 100	> 100
CH_3_-CONH-LGIKILWSARKFGKVIEKSF	16	2321,16	> 100	> 100	> 100	> 100
CH_3_-CONH-LLGIKILWSARKFGKVIEKSF	17	2434,34	> 100	> 100	> 100	> 100
CH_3_-CONH-LLGIKILWKARKFGKVIEKSF	18	2475,44	> 100	> 100	100	> 100
CH_3_-CONH-KILWSAYKHR	S1	1342,59	> 100	> 100	> 100	> 100
CH_3_-CONH-ILWSAYKHR	S2	1214,42	> 100	> 100	> 100	> 100
CH_3_-CONH-LWSAYKHR	S3	1101,26	> 100	> 100	> 100	> 100
CH_3_-CONH-WSAYKHR	S4	988,1	> 100	> 100	> 100	> 100
CH_3_-CONH-WSAYKH	S5	831,92	> 100	> 100	> 100	> 100
CH_3_-CONH-WSAYK	S6	694,78	> 100	> 100	> 100	> 100
H_2_N-LLGDFFRKSKEKIGKEFKRIVQRIKDFLRNLVPRTES	LL-37	4493	> 100	> 100	50	50

The antimicrobial activity was determined against gram-positive *S. aureus (*ATCC 29213, Manassas, VA) and gram-negative *E. coli* (K-12 MG1655) using the broth microdilution method, (peptide diluted in PBS and bacteria in LB-broth, with a final volume of 200 µL, with a ratio of 50:50 PBS/broth). The truncated peptide **6**, which is composed of 16 amino acids, but not full length PLNC8 β, was found to possess inhibitory and bactericidal activity against both *S. aureus* and *E. coli*. The human-derived antimicrobial peptide LL-37 was used as a control.

**Figure 2:**
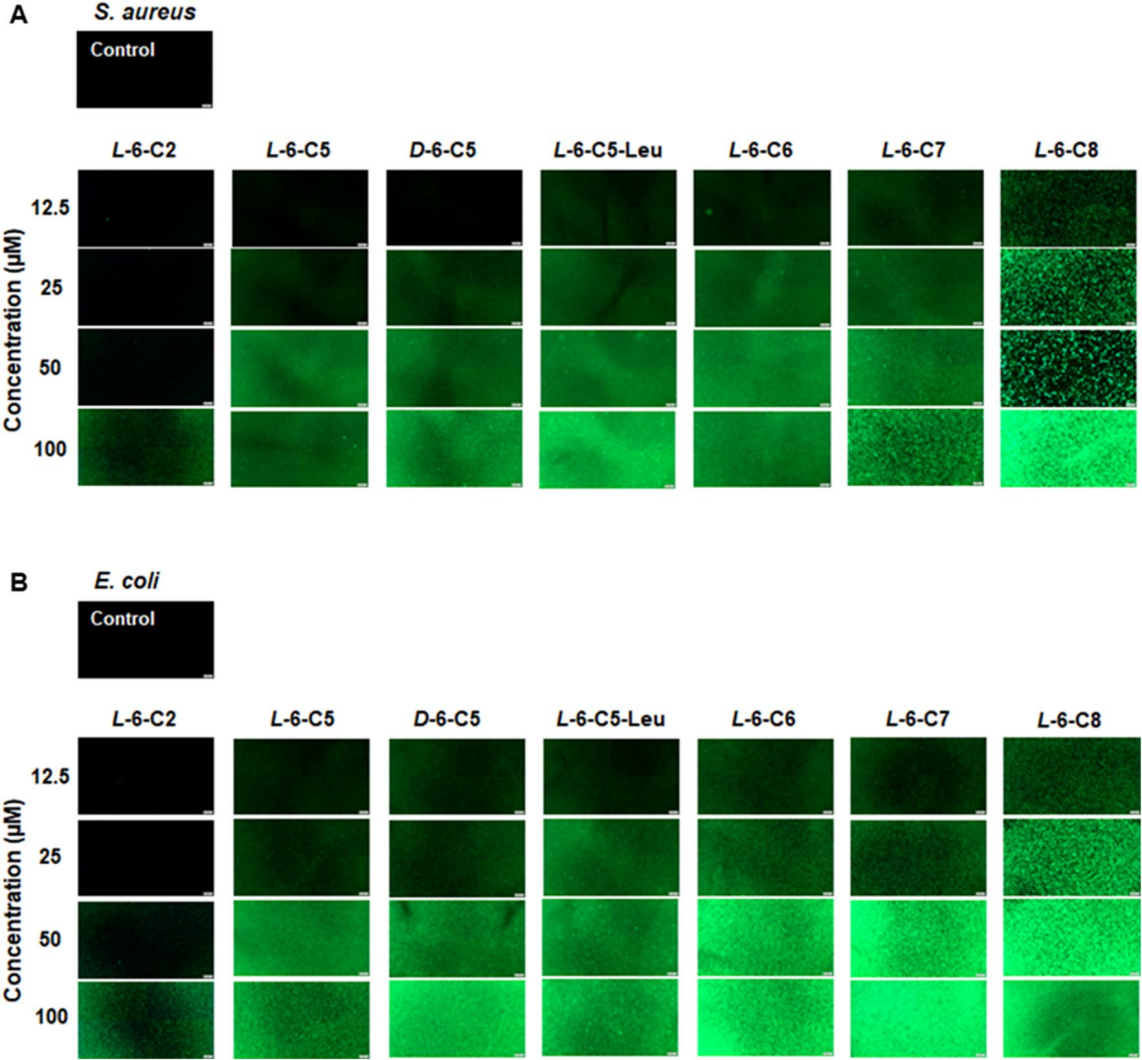
Permeabilization of bacterial membranes. Uptake of Sytox Green by (**A**) *S. aureus* and (**B**) *E. coli* was determined after exposure to different concentrations of acetylated peptides in PBS for 5 min, scale bar is 200 µm. Conjugation of the peptide with a FA chain of five to eight carbon atoms is efficient at permeabilizing both gram-positive and gram-negative bacterial membranes. The *D*-enantiomer and leucine variant of 6-C5 were equally as potent as the *L*-form at permeabilizing *S. aureus* and *E. coli*.

Since peptide **6** showed antibacterial activity in vitro for both *S. aureus* and *E. coli*, additional modifications to improve its stability against proteolytic degradation, and antimicrobial activity, including FA-conjugation and PEGylation of the N-terminal region, were performed. The conjugation of a hydrophobic moiety, such as a FA, to antimicrobial peptides have been shown to modulate their activity and selectivity. Depending on the peptide and properties of the conjugated moeity, this type of modification can be both beneficial or detrimental, e.g. affecting antimicrobial activity and selectivity for bacterial membranes over eukaryotic cells^27^. PEGylation is used in several pharmacological products, primarily to improve drug half-life by preventing proteolytic degradation but can also have other beneficial effects such as reducing recognition by the immune system^28^. PEGylation did not enhance the antimicrobial activity of peptide **6,** but in contrast, conjugation of a FA chain markedly increased the antimicrobial activity (Table 3). Compared to **6**, the MIC and MBC values decreased proportionally to the chain length of the FA, with an optimum seen at five to seven carbon atoms (*L-*6-C5, *L-*6-C6, and *L-*6-C7). Increasing the FA chain length to eight carbon atoms (*L-*6-C8) did not increase antimicrobial activity further against *S. aureus* whereas a decrease in activity against *E. coli* was observed with an increase in MIC and MBC values from 6.2 µM to 12.5 µM, respectively. A FA chain of ≥ 10 carbon atoms drastically reduced bacterial inhibition and completely abolished the bactericidal effects. Furthermore, the *D*-enantiomers of 6-C5, 6-C6, and 6-C7 showed enhanced antimicrobial activity against both *S. aureus* and *E. coli*. To further promote folding of the lipopeptides an additional modification of *L*/*D*-6-C5, *L*/*D*-6-C6, and *L*/*D*-6-C7 included replacement of all three isoleucine (Ile) residues with leucine (Leu). Leu has higher α-helix propensity than Ile, which we hypothesized could further increase the membrane activity of the lipopeptides. Interestingly, the substitution of the three Ile residues with Leu against *S. aureus* reduced the MIC and MBC against *S. aureus* with about 50% compared to the Ile-containing variants. Fluorescence microscopy using a SYTOX Green assay showed that the membrane lytic effect of all the lipopeptides was rapid, with substantial bacterial lysis already after 5 min in a dose-dependent manner (Fig. 2). A more pronounced lysis was observed for lipopeptides modified with FA chains with five to seven carbons compared to those with two or eight carbons.

**Table 3:** Antimicrobial activity and therapeutic indexes of modified variants of peptide sequence 6.

Sequence	Name	MW	*S. aureus*		*E. coli*			*S. aureus*	*E. coli*
			MIC	MBC	MIC	MBC	HC^a^	TI^b^	TI^b^
H_2_N-SVPTSVYTLGIKILWSAYKHRKTIEKSFNKGFYH	*L*-β	4000.6	> 100	> 100	> 100	> 100			
H_2_N-LGIKILWSAYKHRKTI	*L*-6	1926.35	> 100	> 100	> 100	> 100			
CH_3_-CONH-LGIKILWSAYKHRKTI	*L*-6-**C2**	1967.18	100	100	50	50			
CH_3_-(CH_2_)_2_-CONH-LGIKILWSAYKHRKTI	*L*-6-**C4**	1996.46	12.5	25	12.5	25			
CH_3_-(CH_2_)_3_-CONH-LGIKILWSAYKHRKTI	*L*-6-**C5**	2010.48	6.2	6.2	6.2	6.2	> 100	32.3	32.3
CH_3_-(CH_2_)_3_-CONH-LGIKILWSAYKHRKTI	*D*-6-**C5**	2010.48	3.1	6.2	3.1	3.1	> 100	64.5	64.5
CH_3_-(CH_2_)_3_-CONH-LGLKLLWSAYKHRKTL	*L*-6-**C5**-Leu	2010.48	3.1	3.1	6.2	6.2	> 100	64.5	32.3
CH_3_-(CH_2_)_3_-CONH-LGLKLLWSAYKHRKTL	*D*-6-**C5**-Leu	2010.48	3.1	3.1	3.1	3.1	> 100	64.5	64.5
CH_3_-(CH_2_)_4_-CONH-LGIKILWSAYKHRKTI	*L*-6-**C6**	2024.51	3.1	6.2	6.2	6.2	> 100	64.5	32.3
CH_3_-(CH_2_)_4_-CONH-LGIKILWSAYKHRKTI	*D*-6-**C6**	2024.51	3.1	3.1	3.1	3.1	> 100	64.5	64.5
CH_3_-(CH_2_)_4_-CONH-LGLKLLWSAYKHRKTL	*L*-6-**C6**-Leu	2024.51	1.5	3.1	6.2	6.2	60	40	9.7
CH_3_-(CH_2_)_4_-CONH-LGLKLLWSAYKHRKTL	*D*-6-**C6**-Leu	2024.51	1.5	1.5	3.1	3.1	65	43.3	21
CH_3_-(CH_2_)_5_-CONH-LGIKILWSAYKHRKTI	*L*-6-**C7**	2038,53	3.1	3.1	6.2	6.2	80	25.8	13
CH_3_-(CH_2_)_5_-CONH-LGIKILWSAYKHRKTI	*D*-6-**C7**	2038,53	1.5	3.1	3.1	6.2	65	21	21
CH_3_-(CH_2_)_5_-CONH-LGLKLLWSAYKHRKTL	*L*-6-**C7**-Leu	2038,53	1.5	1.5	6.2	6.2	30	20	4.8
CH_3_-(CH_2_)_5_-CONH-LGLKLLWSAYKHRKTL	*D*-6-**C7**-Leu	2038,53	1.5	1.5	3.1	3.1	35	23.3	11.3
CH_3_-(CH_2_)_6_-CONH-LGIKILWSAYKHRKTI	*L*-6-**C8**	2052,55	3.1	3.1	12.5	12.5			
CH_3_-(CH_2_)_8_-CONH-LGIKILWSAYKHRKTI	*L*-6-**C10**	2080.61	50	> 100	> 100	> 100			
CH_3_-(CH_2_)_13_-CONH-LGIKILWSAYKHRKTI	*L*-6-**C15**	2150.75	> 100	> 100	> 100	> 100			
PEG800-LGIKILWSAYKHRKTI	*L*-6-**Peg800**	2708.35	> 100	> 100	> 100	> 100			

The amino-terminal region of the truncated peptide was either left unmodified, acetylated or pegylated. The antimicrobial activities were determined against *S. aureus* (ATCC 29213, Manassas, VA) and *E. coli* (K-12 MG1655), using the broth microdilution method (peptide diluted in PBS and bacteria in LB-broth, with a final volume of 200 µL, with a ratio of 50:50 PBS/broth). Acetylation with five to seven carbon atoms substantially enhanced the inhibitory and bactericidal activity of the truncated peptide against both bacteria. Full length PLNC8 β was used for comparison and as a control. The *D*-enantiomers of 6-C(5–7) showed an enhanced inhibitory and bactericidal activity. Interestingly, replacement of all three isoleucine residues with leucine in 6-C5 improved the antimicrobial activity of both *L*- and *D*-enantiomers against *S. aureus*. ^a^Hemolytic concentration at 5% hemolytic activity (µM). If the cut-off (5% hemolytic activity) was not reached at the highest tested concentration (100 µM), 200 µM was used for calculating the therapeutic index. ^b^Therapeutic Index. HC (µM) at 5% hemolytic activity/MIC (µM). Larger values indicate greater antimicrobial specificity.

In order to further evaluate the activity of the lipopeptide, the MIC and MBC of the leading compounds (*L*/*D*-6-C5 and *L*/*D*-6-C5-Leu) was determined for additional strains of *S. aureus* and *E. coli*. 18 clinical isolate strains of *S. aureus* and 18 clinical isolate strains of *E. col*i were obtained from the Department of Laboratory Medicine of Örebro University Hospital. Of the *S. aureus* strains nine strains were identified as MSSA and nine as MRSA. For *E. coli*, eight strains were identified as ESBL strains and the remaining 10 *E. coli* strains showed none or only minor antibiotic resistance (Supplementary Table S1). In addition to *S. aureus* and *E. coli*, susceptibility testing for *L*/*D*-6-C5 and *L*/*D*-6-C5-Leu was carried out for clinical isolates of the remaining ESKAPE pathogens (*E. faecium*, *K. pneumoniae*, *A. baumannii*, *P. aeruginosa* and *E. cloacae).* All of the tested strains, with the exception of *E. cloacae*, were susceptible to all C5 variants. *E. cloacae*, while susceptible to the *D*-enantiomers of 6-C5 and 6-C5-Leu, was not completely inhibited by the *L*-enantiomers, although some inhibition could be observed. The MIC and MBC of these bacteria is shown in Table 4.

**Table 4:** Susceptibility of clinical strains of *S. aureus*, *E. coli* and the ESKAPE pathogens to *L*/*D*-6-C5 and *L*/*D*-6-C5-Leu.

		MIC µM				MBC µM			
		*L*-6-C5	*D*-6-C5	*L*-6-C5-Leu	*D-6-*C5-Leu	*L*-6-C5	*D*-6-C5	*L*-6-C5-Leu	*D-6-*C5-Leu
*S. aureus*									
505018179796		6.3	3.1	6.3	6.3	12.5	3.1	6.3	6.3
505018186054		6.3	3.1	6.3	3.1	12.5	6.3	6.3	3.1
505018193648		6.3	3.1	6.3	6.3	12.5	6.3	6.3	6.3
505018190785		12.5	3.1	6.3	6.3	25	12.5	6.3	6.3
505018194921		6.3	1.6	3.1	3.1	12.5	6.3	12.5	6.3
YSAR-21-2843		12.5	3.1	6.3	6.3	25	6.3	6.3	6.3
DSAR-21-1211		12.5	3.1	3.1	3.1	12.5	6.3	6.3	6.3
YSAR-21-4907		12.5	3.1	6.3	6.3	12.5	6.3	6.3	6.3
YSAR-21-4909		12.5	6.3	6.3	6.3	25	12.5	12.5	6.3
505017845260	MRSA	12.5	6.3	6.3	6.3	12.5	6.3	6.3	6.3
505018733346	MRSA	12.5	3.1	6.3	6.3	25	3.1	6.3	6.3
505019733436	MRSA	6.3	3.1	3.1	3.1	12.5	3.1	6.3	6.3
505019741940	MRSA	6.3	3.1	3.1	3.1	12.5	3.1	6.3	6.3
505019945723	MRSA	12.5	6.3	6.3	6.3	50	6.3	12.5	6.3
YSAR-21-5398	MRSA	6.3	3.1	6.3	3.1	6.3	3.1	6.3	6.3
YSAR-20-4572	MRSA	6.3	3.1	3.1	3.1	6.3	3.1	6.3	6.3
YSAR-20-5634	MRSA	12.5	6.3	6.3	3.1	12.5	6.3	6.3	12.5
MRSS-20-1637	MRSA	12.5	3.1	6.3	6.3	12.5	3.1	6.3	6.3
*E. coli*									
1250800204		6.3	3.1	6.3	1.6	12.5	3.1	12.5	1.6
1257800639		12.5	3.1	6.3	1.6	25	3.1	12.5	3.1
1257800519		12.5	3.1	6.3	3.1	12.5	3.1	12.5	6.3
1157808982		12.5	3.1	12.5	1.6	25	3.1	12.5	6.3
1250800910		6.3	3.1	6.3	1.6	25	3.1	6.3	3.1
1257800244		6.3	3.1	12.5	1.6	25	3.1	12.5	1.6
1150822152		12.5	3.1	12.5	1.6	25	3.1	12.5	1.6
1257800301		6.3	3.1	6.3	0.8	12.5	3.1	12.5	1.6
1257800601		6.3	1.6	6.3	3.1	12.5	3.1	6.3	3.1
1157809008		6.3	3.1	6.3	1.6	6.3	3.1	6.3	1.6
07T-0846	ESBL	6.3	3.1	3.1	0.8	12.5	3.1	6.3	1.6
07T-1105	ESBL	3.1	1.6	3.1	0.8	6.3	1.6	6.3	0.8
08T-0189	ESBL	3.1	1.6	3.1	1.6	6.3	1.6	3.1	1.6
08T-0315	ESBL	6.3	3.1	3.1	3.1	12.5	6.3	6.3	3.1
08T-0855	ESBL	6.3	3.1	6.3	3.1	12.5	3.1	6.3	3.1
09B-0005	ESBL	3.1	1.6	6.3	3.1	12.5	1.6	12.5	3.1
07T-1294	ESBL	3.1	1.6	3.1	1.6	12.5	6.3	6.3	1.6
07T-0246	ESBL	6.3	3.1	12.5	3.1	12.5	3.1	12.5	3.1
*E. faecium*		6.3	3.1	3.1	3.1	12.5	3.1	6.3	3.1
*K. pneumoniae*		50	25	25	12.5	100	50	50	25
*A. baumannii*		3.1	3.1	3.1	3.1	6.3	3.1	3.1	3.1
*P. aeruginosa*		50	25	25	12.5	100	50	50	25
*E. cloacae*		> 100	25	> 100	25	> 100	50	> 100	50

The antimicrobial activity of *L*/*D*-6-C5 and *L*/*D*-6-C5-Leu was tested against clinical isolates of *S. aureus* (MSSA), *S. aureus* (MRSA), *E. coli*, *E. faecium, K. pneumoniae*, *A. baumannii*, *P. aeruginosa* and *E. cloacae* using the broth microdilution method (peptide diluted in PBS and bacteria in LB-broth, with a final volume of 200 µL, with a ratio of 50:50 PBS/broth). Antibiotic resistance patterns and harvest locale are shown in Supplementary Table S1.

**Figure 3:**
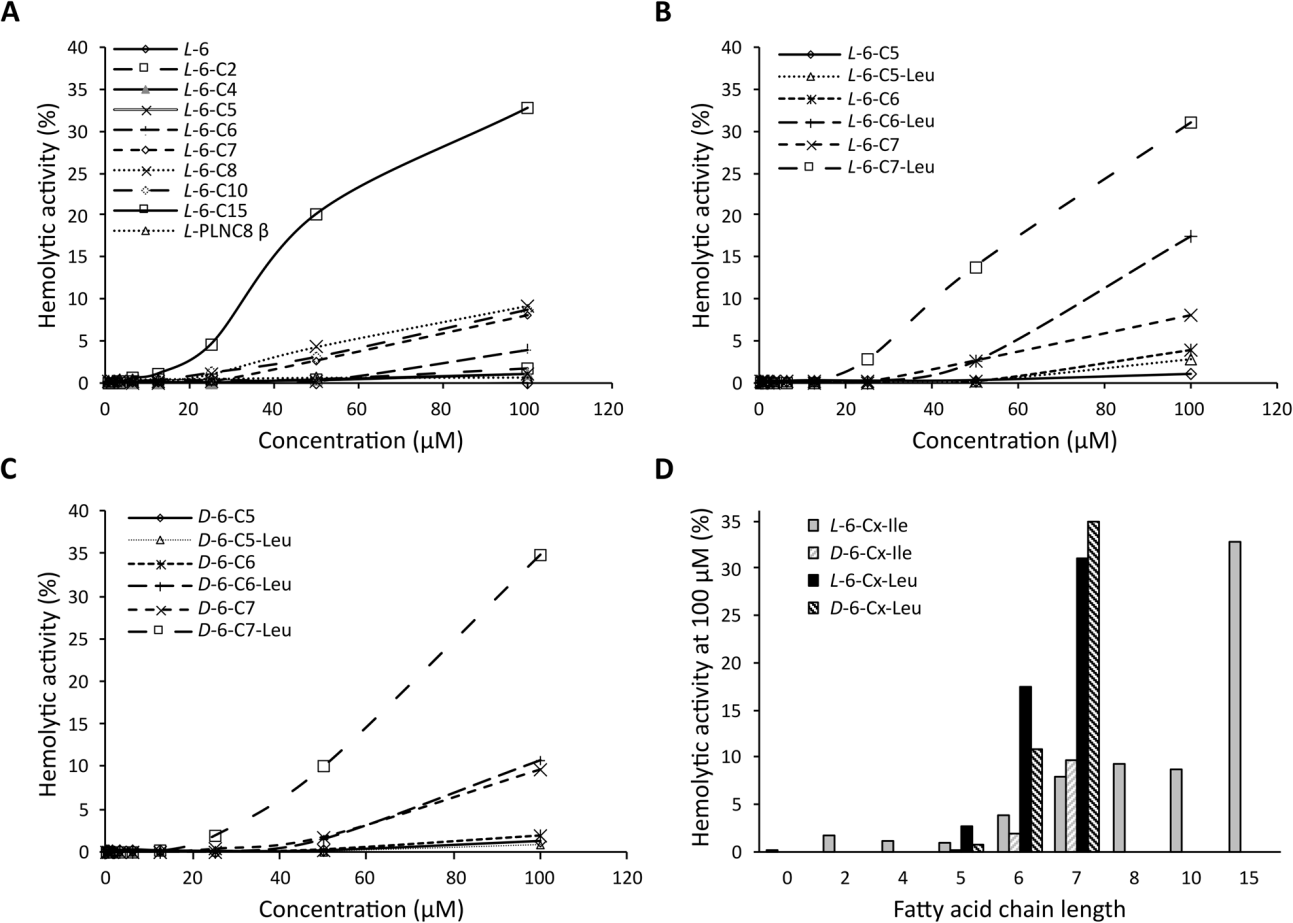
Hemolytic activity of full length and modified peptides of PLNC8 β. The hemolytic activity was determined on human erythrocytes (15% suspended in PBS) after (**A**) exposure to the peptides with different lengths of FA chains using the indicated concentrations for 1 h. Hemolytic activity was associated with the length of the FA chain, where conjugation of long FA chain (≥ 7 carbon atoms) rendered the peptides to be more hemolytic. Hemolytic activity of the *L*-enantiomers (**B**) and *D*-enantiomer (**C**) of 6-C(5–7) of both isoleucine and leucine variants. Replacement of all three isoleucine residues with leucine rendered the lipopeptide to be more hemolytic. (**D**) Hemolytic activity at 100 µM in regard to FA chain length.

Cytotoxicity and hemolytic activity of synthetic AMPs has been problematic and is an important aspect to carefully evaluate. Hemolytic activity was found to be associated with the length of the FA chain (Fig. 3A). Lipopeptides with a FA chain length with ≤ 6 carbon atoms caused less than 5% hemolysis at 100 µM after 1 h incubation with erythrocytes in PBS at 37 °C. Lipopeptides with FA chains with 7–10 carbon atoms caused about 10% hemolysis. Increasing the length of the FA chain to 15 carbon atoms increased the hemolysis to 40%. The *L*-enantiomers of 6-C5, 6-C6, and 6-C7 showed higher hemolytic activity than the corresponding *D*-enantiomer, reaching ~ 10%. An additional increase in hemolytic activity was seen for the Leu-containing variants (Fig. 3B). The *D*-enantiomers of 6-C5, 6-C6, and 6-C7 showed similar hemolytic activity as the *L*-forms (Fig. 3C). Hemolytic activity at 100 µM in relation to FA chain length is shown in Fig. 3D.

The membrane activity of the lipopeptides was further confirmed using liposome models with either prokaryotic or eukaryotic mimicking lipid compositions. The ability of the lipopeptides to perturb lipid membrane integrity was investigated by monitoring the release of the fluorescent dye carboxyfluorescein (CF) encapsulated in the liposomes at self-quenching concentrations (Supplementary Fig. S3). The permeabilization of bacterial-mimicking liposomes was observed to take place at lipopeptide concentration < 1 µM for all sequences with a FA chain length of eight or fewer carbon atoms (Supplementary Fig. S3A). Increasing the length of the FA chain resulted in a decrease in membrane activity (Supplementary Fig. S3B). Full length PLNC8 β and the human-derived antimicrobial peptide LL-37 were used as controls (Supplementary Fig. S3F). The CF release was slower for the lipopeptides compared to full length PLNC8 β. The latter showed permeabilization of liposome membranes even at concentrations as low as 100 pM, resulting in ~ 20% CF release after 60 min of incubation (Supplementary Fig. S4). Membrane activity of LL-37 was inferior to all lipopeptides with a FA chain with fewer than eight carbon atoms (Table 5).

**Table 5:** Membrane activity of lipopeptides using liposome model system.

Label	EC50 (µM)	
	Bacterial	Mammalial
*L*-6	0.15	> 100
*L*-6-C2	0.11	> 100
*L*-6-C4	0.11	> 10
*L*-6-C5	0.11	10.45
*D*-6-C5	0.12	13.07
*L*-6-C5-Leu	0.11	3.99
*D*-6-C5-Leu	0.13	4.08
*L*-6-C6	0.16	3.57
*D*-6-C6	0.13	4.99
*L*-6-C6-Leu	0.13	1.21
*D*-6-C6-Leu	0.13	1.22
*L*-6-C7	0.22	1.95
*D*-6-C7	0.29	1.47
*L*-6-C7-Leu	0.12	1.10
*D*-6-C7-Leu	0.13	1.02
*L*-6-C8	0.19	0.94
*L*-6-C10	1.10	0.68
*L*-6-C15	19.40	0.87
LL-37	0.96	0.68
*L*-PLNC8 β	0.02	1.26

The membrane activity of the modified variants of peptide sequence **6** was determined using a bacterial membrane-mimicking liposome model (75:25 POPC:POPG) and a mammalian membrane-mimicking liposome model (65:5:30 POPC:POPS:Chol.). Full length PLNC8 β and human-derived antimicrobial peptide LL-37 were used as controls. 10^–5^–10^2^ µM lipopeptides were incubated with 25 µM liposome models in 10 mM PBS buffer (pH 7.4). The concentration that triggers 50% CF release (EC50) was determined after 60 min of incubation.

Membrane activity was further evaluated towards mammalian-lipid membrane mimicking liposomes. Permeabilization effect was measured to be roughly 10- to 100-fold higher if compared to bacterial mimicking liposomes, demonstrating the suitability of these lipopeptides for use in bacterial targeting (Table 5). Membrane activity increased with FA chain length up to 8 carbon atoms, after which no further increase was seen (Supplementary Fig. S3G,H).

Irrespectively of lipid composition, both the *L*- and *D*- enantiomers of the lipopeptides had similar membrane activity and CF release kinetics (Supplementary Figs. S3C–E,I–K, S4, S5). Surprisingly, the substitution of isoleucine for leucine resulted in a slight increase in membrane activity in both bacterial and mammalian lipid membrane-mimicking liposomes, with the most pronounced effect in the latter (Table 5).

**Figure 4:**
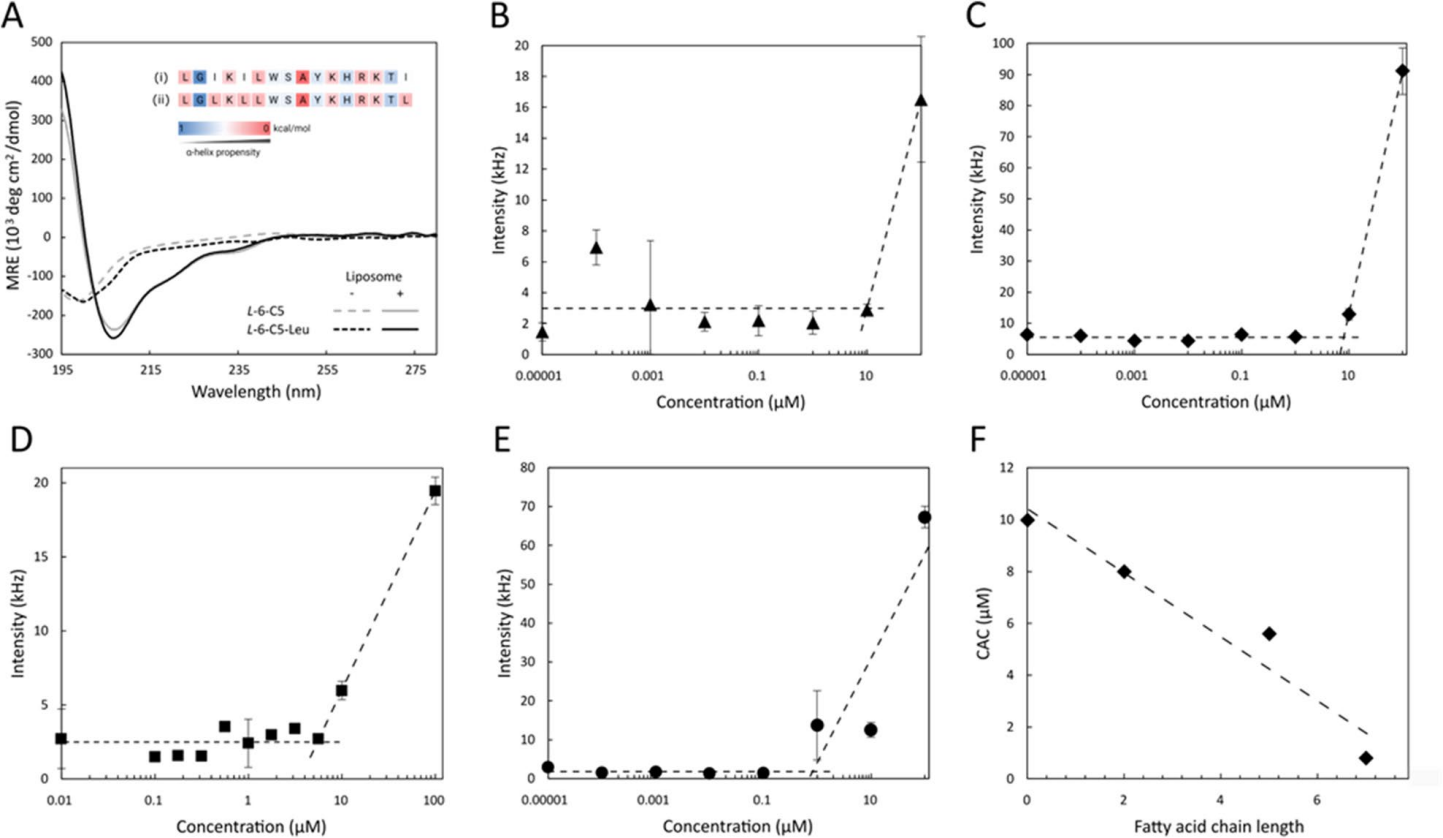
Secondary structure characterization and critical aggregation concentration (CAC) determination. (**A**) CD spectra of 30 µM *L*-6-C5 and *L*-6-C5-Leu peptides without (dotted line) and with (full line) 1.2 mM POPC:POPG (75:25) liposome models in PBS buffer (10 mM, pH 7.4). Inset: analysis of amino acid propensity of (i) *L*-6-C5 and (ii) *L*-6-C5-Leu peptide sequences (with exclusion of the fatty acid tail). (**B-E**) DLS analysis: scattering intensity of lipopeptides (**B**) *L*-6, (**C**) *L*-6-C2, (**D**) *L*-6-C5 and (**E**) *L*-6-C7 in 10 mM PBS (pH 7.4). (**F)** Relationship between critical aggregation concentration (CAC) and fatty acid chain length of the lipopeptides *L*-6, *L*-6-C2, *L*-6-C5 and *L*-6-C7.

Since the lipopeptides are amphipathic they may be prone to self-assemble into larger supramolecular structures, which could influence membrane activity. We employed dynamic light scattering (DLS) to study lipopeptide aggregation and the dependency on FA chain length on their solubility, focusing on the peptides *L*-6, *L*-6-C2, *L*-6-C5 and *L*-6-C7. The amphiphilic character of lipopeptides determines their propensity to self-assemble in aqueous solutions. We can thus hypothesize that the lipopeptides form micelle-like structures that are in dynamic equilibrium with dissolved lipopeptide monomers. Above the critical aggregation concentration (CAC), the formation of larger aggregates is favored, at the expense of dissolved lipopeptides, resulting in an increase in scattering intensity in the DLS. The CAC of the lipopeptides *L*-6, *L*-6-C2, *L*-6-C5 and *L*-6-C7 was estimated to 10, 8, 5.6 and 0.8 μM, respectively (Fig. 4B–F), highlighting the correlation between the FA chain length and the propensity to aggregate.

AMPs are typically random coils in solution but fold upon interaction with bacterial membranes. The secondary structure of the lipopeptides *L*-6-C5 and *L*-6-C5-Leu was evaluated using circular dichroism (CD) spectroscopy (Fig. 4A) in the absence and presence of bacterial-mimicking liposome models composed of POPC:POPG (75:25). Both lipopeptides were predominantly random coil in solution in the absence of lipid membranes. However, we observed a pronounced change in lipopeptide secondary structure when interacting with liposomes, partly indicating presence of α-helical secondary structure elements. The lipopeptide *L*-6-C5 presents a homogeneous mix of amino acids with both a-helix and β-strand propensities, as well as two structure-breaking residues^29^, Gly and Ser in position 2 and 8, respectively. A well-defined secondary structure is thus not expected, neither in solution nor when interacting with lipid membranes. The exchange of Ile for Leu in position 3, 5 and 16 yielding the lipopeptide *L-*6-C5-Leu, increase the number of amino acids with high α-helix propensity from 7 to 10 (Fig. 4A,ii)^30^. Despite having very similar hydrophobicity, Leu (0.21 kcal/mol) is a significantly better helix former than Ile (0.41 kcal/mol). However, although this modification improved the MIC and MBC values, no significant changes in secondary structure were observed, likely because of the clustering of the leucine residues close to the peptide N-terminus (Leu-Lys-Leu-Leu in position 3–6), which is flanked by Gly in position 2 and Ser in position 8, which destabilizes the helical structure.

A major concern when developing new antibacterial compounds is the risk of resistance development. Both *S. aureus* and *E. coli* cultured in the presence of sub-MIC concentrations of *L*-6-C5 for 30 passages retained the same MIC- and MBC-values as the unexposed bacteria (passage 0), indicating no development of resistance (Supplementary Table S2).

The *L*- and *D*-enantiomers of the lipopeptide 6-C5 and *L*- and *D*-enantiomers of the variants of 6-C5-Leu with isoleucine substituted for leucine were combined with different antibiotics and the antimicrobial activity was determined against *E. coli* and *S. aureus*. The lipopeptides were able to decrease the concentrations of antibiotics required to inhibit and kill the bacteria in an additive or synergistic manner in most of the combinations tested, although a few combinations exhibit an indifferent relationship. Importantly, none of the combinations showed any signs of being antagonistic (Table 6).

**Table 6:** Antimicrobial activity of acetylated peptides in combination with antibiotics.

Antimicrobial agent	*S. aureus*			Antimicrobial agent	*E. coli*		
	MIC	MBC	ΣFIC/FBC		MIC	MBC	ΣFIC/FBC
***L-*6-C5 (µM)**	**6.25**	**6.25**		***L-*6-C5 (µM)**	**6.25**	**6.25**	
***D-*6-C5 (µM)**	**3.1**	**6.25**		***D-*6-C5 (µM)**	**3.1**	**6.25**	
***L-*6-C5-Leu (µM)**	**3.1**	**3.1**		***L-*6-C5-Leu (µM)**	**3.1**	**3.1**	
***D-*6-C5-Leu (µM)**	**3.1**	**3.1**		***D-*6-C5-Leu (µM)**	**3.1**	**3.1**	
**Vancomycin (µg/ml)**	**0.78**	**0.78**		**Gentamicin (µg/ml)**	**1**	**1**	
Vancomycin/*L*-6-C5			**0.75/1.00**	Gentamicin/*L*-6-C5			**0.75/1.00**
Vancomycin/*D-*6-C5			**0.82/0.70**	Gentamicin/*D-*6-C5			**0.52/0.63**
Vancomycin/*L*-6-C5-Leu			**0.70/0.75**	Gentamicin/*L*-6-C5-Leu			**0.75/0.75**
Vancomycin/*D-*6-C5-Leu			**0.70/0.78**	Gentamicin/*D-*6-C5-Leu			**0.51/0.51**
**Rifampicin (µg/ml)**	**0.062**	**0.062**		**Rifampicin (µg/ml)**	**12.5**	**100**	
Rifampicin/*L*-6-C5			**1.06/1.06**	Rifampicin/*L*-6-C5			**1.06/1.06**
Rifampicin/*D-*6-C5			**1.03/1.00**	Rifampicin/*D-*6-C5			**0.53/1.00**
Rifampicin/*L*-6-C5-Leu			**1.06/1.13**	Rifampicin/*L*-6-C5-Leu			**0.53/0.50**
Rifampicin/*D-*6-C5-Leu			**1.03/1.02**	Rifampicin/*D-*6-C5-Leu			**0.56/0.51**
**Tetracycline (µg/ml)**	**0.5**	**8**		**Cefotaxim (µg/ml)**	**0.5**	**0.5**	
Tetracycline/*L*-6-C5			**0.53/0.63**	Cefotaxim/*L*-6-C5			**0.53/0.63**
Tetracycline/*D-*6-C5			**0.75/0.75**	Cefotaxim/*D-*6-C5			**0.75/0.75**
Tetracycline/*L*-6-C5-Leu			**0.63/0.53**	Cefotaxim/*L*-6-C5-Leu			**0.53/0.56**
Tetracycline/*D-*6-C5-Leu			**0.53/0.53**	Cefotaxim/*D-*6-C5-Leu			**0.53/0.56**
**Ciprofloxacin (µg/ml)**	**0.25**	**0.25**		**Ciprofloxacin (µg/ml)**	**0.125**	**0.125**	
Ciprofloxacin/*L*-6-C5			**0.53/0.37**	Ciprofloxacin/*L*-6-C5			**0.53/0.37**
Ciprofloxacin/*D-*6-C5			**0.76/0.75**	Ciprofloxacin/*D-*6-C5			**0.63/0.63**
Ciprofloxacin/*L*-6-C5-Leu			**0.63/0.56**	Ciprofloxacin/*L*-6-C5-Leu			**0.63/0.56**
Ciprofloxacin/*D-*6-C5-Leu			**0.63/0.63**	Ciprofloxacin/*D-*6-C5-Leu			**0.63/0.63 s**

The antimicrobial activities of acetylated peptides with five carbon atoms, with or without different antibiotics, were determined in checkerboard assays (peptide diluted in PBS and bacteria in LB-broth, with a final volume of 200 µL, with a ratio of 50:50 PBS/broth) against (**A**) *S. aureus* (ATCC 29213, Manassas, VA) and (**B**) *E. coli *(K-12 MG1655). The fractional inhibitory concentration (FIC) and fractional bactericidal concentration (FBC) shows that the majority peptides interact with the antibiotics in a synergistic or additive manner. Synergy was defined as FIC/FBC ≤ 0.5, additive when 0.5 < FIC/FBC ≤ 1, indifferent when 1 < FIC/FBC < 2 and antagonistic when FIC/FBC ≥ 2.

## Discussion

We have recently shown that the antimicrobial activity of the bacteriocin PLNC8 αβ against *Staphylococcus* spp., is optimal when both the α and β peptides are used together in a molar ratio of 1:1^14^. PLNC8 αβ is an efficient and valuable bacteriocin against gram-positive bacteria, however it would be advantageous to develop short and optimized peptides of PLNC8 αβ with broad spectrum activity to also target challenging infections caused by gram-negative bacteria. PLNC8 β, but not PLNC8 α, showed lytic activity on liposomes and against bacteria, but it was not sufficient to inhibit bacterial growth. Development of short and optimized antimicrobial peptides was therefore based on the amino acid sequence of PLNC8 β.

We were able to identify and develop several novel compounds that exhibit high antimicrobial activity in vitro, even at very low doses. Chu-Kung and colleagues^27^ showed an increased antimicrobial activity of peptides following conjugation of lauric acid (FA chain with a 12 carbon atom backbone). Our results in obtaining a significantly improved antimicrobial activity after conjugation of short FA chain (five to seven carbon atoms) are thus surprising. Laverty et al., 2010^31^ analyzed a range of lipopeptides with different lengths of FAs, and showed that the antimicrobial activity was enhanced by increasing the length of the FA chain. The most effective variant comprised a FA chain with 12 carbon atoms, which is in line with the results obtained by Chu-Kung and colleagues^27^. However, this lipopeptide was shown to be highly hemolytic and cytotoxic at concentrations ≥ 50 µg/ml, resulting in complete hemolysis. Our results also showed increased hemolytic activity with increased FA length, but since the antibacterial activity was reduced in our lipopeptides with FA chains comprised of 8 carbons or more, the lipopeptides conjugated with the shorter FA chain of 5–7 carbons were thus considered the prime candidates for further examination. Furthermore, the *D*-enantiomers of 6-C5, 6-C6, and 6-C7 showed enhanced antimicrobial activity against both *S. aureus* and *E. coli*, which is likely due to resistance against proteolytic degradation. The same trend could be observed for the ESKAPE pathogens, where the *D*-enantiomers displayed better activity than the *L*-enantiomers, especially for *K. pneumoniae*, *P. aeruginosa* and *E. cloacae*. The most prominent difference was observed in *E. cloacae*, where the highest concentration of the *L*-enantiomers tested (100 µM) was not sufficient to fully inhibit and kill the bacteria, suggesting increased proteolytic activity among some of these strains, which is in line with reported pathogenicity and resistance among clinical isolates of the ESKAPE pathogens.^32^.

In addition, the substitution of isoleucine with leucine further enhanced the antimicrobial activity of the lipopeptide. As mentioned in the results, leucine has a higher propensity for α-helical formation than isoleucine, which might be part of the explanation to the increased activity, although no significant change in secondary structure was observed. In addition, the single substitution of leucine with isoleucine or vice versa have previously been reported to alter the function of proteins and antibodies^33,34^, which could explain the increased antimicrobial activity observed in our results, but the exact mechanism remains to be determined. These results indicate that the peptide does not recognize and bind to specific target molecules, e.g. proteins and glycoproteins on the bacterial surface, suggesting that the initial binding is driven by electrostatic interactions with anionic bacterial structures, such as membrane lipids, which indeed is one of the common mechanisms of action in AMPs^35^. Although the precise mechanisms remain to be determined, including initial interactions with the bacterial cell wall (gram-positive) or outer membrane (gram-negative), the rapid permeabilization indicates that the final and main target of the lipopeptides is indeed bacterial membranes.

Dynamic light scattering studies allowed to highlight the role of lipopeptide aggregation in the process of bacterial membrane permeabilization. The aggregation of the lipopeptides *L*-6, *L*-6-C2, *L*-6-C5 and *L*-6-C7 was evaluated in buffer, demonstrating a decrease in critical aggregation concentration (CAC) with an increase in carbon tail length, with CACs corresponding to 10 µM, 8 µM, 5.6 µM and 0.8 µM, respectively (Fig. 4 B-F). When comparing CAC values with MBC, we found the latter to be higher than CAC against both gram-positive *S. aureus* (> 100 μM, 100 μM, 6.2 μM and 3.1 μM relatively to the lipopeptides *L*-6, *L*-6-C2, *L*-6-C5 and *L*-6-C7, respectively) and gram-negative *E. coli* (> 100 μM, 50 μM, 6.2 μM and 6.2 μM, relatively to the lipopeptides *L*-6, *L*-6-C2, *L*-6-C5 and *L*-6-C7 respectively). These findings strongly indicate that formation of lipopeptide aggregates contribute to their antimicrobial activity.

Although the exact mechanism of action is still unknown, some conclusions can be drawn. Lipopeptides are well known to spontaneously interact and organize, forming micelle-like structures owing to their amphiphilic nature^36,37^. The initial driving force for the association with bacterial membrane is represented by electrostatic interaction, as mentioned above. Attraction between the cationic residues (Arg and Lys, in position 13 and 4, 11, 14 respectively) and the negatively charged phosphate groups of the bacterial membrane stabilize the interactions through hydrogen bonds^37,38^. A second event driving the lipopeptide association with the bacterial membrane is constituted by the hydrophobic effect and van der Waals interactions between the lipopeptide acyl chains and the hydrophobic core of the bacterial membrane resulting in lipid-membrane partitioning. Ascribed to poor micelle packaging^36^ or to ordering disruption of the same as a consequence of the initial micelle-membrane binding^37^, exposed lipid tails become available for interaction, resulting in aggregate dissociation and lipopeptide insertion into the lipid bilayer. Although it is still unknown whether the aggregates or the individual lipopeptides contributes most to cell lysis, it appears certain that the aggregate accumulation at the bacterial membrane surface drastically increases the local concentration of lipopeptides, resulting in efficient membrane disruption^38^. As indicated by CD spectroscopy, these interactions did not trigger any changes in lipopeptide secondary structure, which is otherwise very common for AMPs.

In addition to their inherent ability to kill bacteria or inhibit bacterial growth, antimicrobial peptides that target bacterial membranes are also attractive candidates for use in combination with more conventional antibiotics to increase their efficacy and to suppress the development and spreading of antimicrobial resistance. Nisin has been shown to act synergistically with citric acid^39^, penicillin, and chloramphenicol^40^ against several *Staphylococcus* species. We have previously shown that plantaricin A, E, F, J, K^41^, and NC8 αβ^14^ substantially enhance the effects of several antibiotics, including vancomycin, teicoplanin, rifampicin, gentamicin, and tetracycline, against *Staphylococcus* species. Our results further suggest that the novel lipopeptides presented here may also be used in combination with antibiotics. Combination therapy has been utilized to reduce the required concentration of antibiotics, and consequently reduce possible side-effects, environmental contaminations, and development of resistance.

The ease with which bacteria can develop resistance towards antibiotics is one of the reasons so few new classes of antibiotics have been made available for clinical use in the last decades. Very few large pharmacological companies remain active in the field of antibiotics discovery, a field which today is mostly pursued in smaller academic labs. The reason is mainly a financial issue; because of risks associated with the development of resistance to new antibiotic compounds, the field of antibiotics is not as profitable as several other fields of drug development^42,43^. Although the risks of development of bacterial resistance to AMPs have long been considered small, something that has been a contributing factor in the increased interest in research regarding AMPs, reports of AMP resistance are increasing. Some of the bacterial mechanisms of resistance to AMPs include the use of proteolytic enzymes and membrane alterations resulting in a change in net charge thus limiting the ability of the peptide to interact with the membrane^44^. One must therefore consider the possibility of resistance development also in research regarding AMPs. Our results showed no indications of resistance being developed in either bacterium tested, when exposed to sub-MIC concentrations of the lipopeptide for 30 passages, again suggesting that the evolutionary conserved lipid membranes of the bacteria constitute the target of the peptide. As mentioned above, the use of *D-*enantiomers can also be a strategy to combat degradation by proteases, although it is unclear how this could affect toxicity and accumulation, especially considering systemic administration. Nevertheless, given enough time it is likely that some resistance will be developed, and as with all antibacterial substances, care must be taken to avoid unnecessary exposure.

While numerous different antimicrobial peptides with excellent effects in vitro have been identified over the past decades, few have made it into clinical practice because of limitations such as proteolytic susceptibility, cytotoxicity, poor bioavailability, and contextual sensitivity, and those that indeed have been made clinically available are most often formulated for topical use^45^. Therefore, bioengineered modifications of AMPs, such as those explored in this paper, have become increasingly relevant to push past said limitations and make AMPs more suited for pharmacological use^46^. While further research is required to determine the potential therapeutic use of these novel lipopeptides, where in vivo studies of lipopeptide toxicity and activity are of special importance, the selectivity of the lipopeptides towards bacterial membranes versus mammalian membranes is a promising aspect.

In conclusion, we have carried out a rational design process to identify a small library of novel lipopeptides derived from Plantaricin NC8 αβ. The lipopeptides exhibit effective antibacterial activity towards both gram-positive and gram-negative bacteria even at micromolar concentrations. When taking into consideration the hemolytic activity and the EC50 presented above, and the relative similarities in antimicrobial activity among the compounds supplemented with FA chains of 5–7 carbon atoms, we suggest that the compounds conjugated with a FA tail of 5 carbon atoms (6-C5 and 6-C5-Leu) are of most interest for further research. Furthermore, the methods for the identification and design of these lipopeptides employed in this study provides additional pathways to pursue in the development of novel antimicrobial compounds. While PLNC8 αβ remains an interesting pharmaceutical candidate, in particular for treatment of infections caused by *Staphylococcus* spp., the lipopeptides presented here have several benefits. Whereas PLNC8 αβ has limited solubility and tends to precipitate under physiological conditions (data not published), the lipopeptides form colloidally stable assemblies. In addition, compared to PLNC8 αβ which is a two-peptide bacteriocin comprising an α and a β peptide with 29 and 34 residues, respectively^47^, the lipopeptides are less complex. Yet, the antibacterial activity of these lipopeptides is significantly higher than for PLNC8 αβ and they are effective towards both gram-positive and gram-negative bacteria which is a highly desired attribute for any new antibacterial compound.

## Supplementary Information

Supplementary Information.

## Data Availability

The datasets generated and/or analyzed during the current study are available from the corresponding author(s) upon request.

## References

[1] Assis LM, Nedeljkovic M, Dessen A (2017). New strategies for targeting and treatment of multi-drug resistant Staphylococcus aureus. Drug Resist. Updates.

[2] Fridkin SK (2003). Epidemiological and microbiological characterization of infections caused by Staphylococcus aureus with reduced susceptibility to vancomycin, United States, 1997–2001. Clin. Infect. Dis..

[3] Kali A (2015). Antibiotics and bioactive natural products in treatment of methicillin resistant Staphylococcus aureus: A brief review. Pharmacogn. Rev..

[4] Lowy FD (2003). Antimicrobial resistance: The example of Staphylococcus aureus. J. Clin. Investig..

[5] Cotter PD, Ross RP, Hill C (2013). Bacteriocins: A viable alternative to antibiotics?. Nat. Rev. Microbiol..

[6] Czaplewski L (2016). Alternatives to antibiotics-a pipeline portfolio review. Lancet. Infect. Dis.

[7] Yang SC, Lin CH, Sung CT, Fang JY (2014). Antibacterial activities of bacteriocins: Application in foods and pharmaceuticals. Front. Microbiol..

[8] Diep DB, Straume D, Kjos M, Torres C, Nes IF (2009). An overview of the mosaic bacteriocin pln loci from Lactobacillus plantarum. Peptides.

[9] Ekblad B (2016). Structure-function analysis of the two-peptide bacteriocin plantaricin EF. Biochemistry.

[10] Oppegard C, Kjos M, Veening JW, Nissen-Meyer J, Kristensen T (2016). A putative amino acid transporter determines sensitivity to the two-peptide bacteriocin plantaricin JK. MicrobiologyOpen.

[11] Moretta A (2021). Antimicrobial peptides: A new hope in biomedical and pharmaceutical fields. Front. Cell. Infect. Microbiol..

[12] Bengtsson T (2017). Dual action of bacteriocin PLNC8 alphabeta through inhibition of Porphyromonas gingivalis infection and promotion of cell proliferation. Pathogens Dis..

[13] Khalaf H (2016). Antibacterial effects of Lactobacillus and bacteriocin PLNC8 alphabeta on the periodontal pathogen Porphyromonas gingivalis. BMC Microbiol..

[14] Bengtsson T (2020). Plantaricin NC8 alphabeta exerts potent antimicrobial activity against Staphylococcus spp. and enhances the effects of antibiotics. Sci. Rep..

[15] Mulani MS, Kamble EE, Kumkar SN, Tawre MS, Pardesi KR (2019). Emerging strategies to combat ESKAPE pathogens in the era of antimicrobial resistance: A review. Front. Microbiol..

[16] Rozek A, Powers JP, Friedrich CL, Hancock RE (2003). Structure-based design of an indolicidin peptide analogue with increased protease stability. Biochemistry.

[17] Lee IH, Cho Y, Lehrer RI (1997). Effects of pH and salinity on the antimicrobial properties of clavanins. Infect. Immun..

[18] Molchanova N, Hansen PR, Franzyk H (2017). Advances in development of antimicrobial peptidomimetics as potential drugs. Molecules.

[19] Deslouches B, Montelaro RC, Urish KL, Di YP (2020). Engineered Cationic Antimicrobial Peptides (eCAPs) to Combat Multidrug-Resistant Bacteria. Pharmaceutics.

[20] Lata S, Sharma BK, Raghava GP (2007). Analysis and prediction of antibacterial peptides. BMC Bioinform..

[21] Lee HT, Lee CC, Yang JR, Lai JZ, Chang KY (2015). A large-scale structural classification of antimicrobial peptides. Biomed. Res. Int..

[22] Shen Y, Maupetit J, Derreumaux P, Tuffery P (2014). Improved PEP-FOLD approach for peptide and miniprotein structure prediction. J. Chem. Theory Comput..

[23] Thevenet P (2012). PEP-FOLD: An updated de novo structure prediction server for both linear and disulfide bonded cyclic peptides. Nucleic Acids Res..

[24] Alland C (2005). RPBS: A web resource for structural bioinformatics. Nucleic Acids Res..

[25] Jenssen H, Hamill P, Hancock RE (2006). Peptide antimicrobial agents. Clin. Microbiol. Rev..

[26] Clark S, Jowitt TA, Harris LK, Knight CG, Dobson CB (2021). The lexicon of antimicrobial peptides: A complete set of arginine and tryptophan sequences. Commun. Biol..

[27] Chu-Kung AF, Nguyen R, Bozzelli KN, Tirrell M (2010). Chain length dependence of antimicrobial peptide-fatty acid conjugate activity. J. Colloid Interface Sci..

[28] Turecek PL, Bossard MJ, Schoetens F, Ivens IA (2016). PEGylation of biopharmaceuticals: A review of chemistry and nonclinical safety information of approved drugs. J. Pharm. Sci..

[29] Imai K, Mitaku S (2005). Mechanisms of secondary structure breakers in soluble proteins. Biophysics.

[30] Pace CN, Scholtz JM (1998). A helix propensity scale based on experimental studies of peptides and proteins. Biophys. J ..

[31] Laverty G, McLaughlin M, Shaw C, Gorman SP, Gilmore BF (2010). Antimicrobial activity of short, synthetic cationic lipopeptides. Chem. Biol. Drug Des..

[32] De Oliveira DMP (2020). Antimicrobial resistance in ESKAPE pathogens. Clin. Microbiol. Rev..

[33] Sitbon M (1991). Substitution of leucine for isoleucine in a sequence highly conserved among retroviral envelope surface glycoproteins attenuates the lytic effect of the Friend murine leukemia virus. Proc. Natl. Acad. Sci. U S A.

[34] Bagal D, Kast E, Cao P (2017). Rapid distinction of leucine and isoleucine in monoclonal antibodies using nanoflow LCMS(n). Anal. Chem..

[35] Hollmann A, Martinez M, Maturana P, Semorile LC, Maffia PC (2018). Antimicrobial peptides: Interaction with model and biological membranes and synergism with chemical antibiotics. Front. Chem..

[36] Horn JN, Romo TD, Grossfield A (2013). Simulating the mechanism of antimicrobial lipopeptides with all-atom molecular dynamics. Biochemistry.

[37] Armas F (2019). Design, antimicrobial activity and mechanism of action of Arg-rich ultra-short cationic lipopeptides. PLoS ONE.

[38] Li J (2017). Membrane active antimicrobial peptides: Translating mechanistic insights to design. Front. Neurosci..

[39] Zhao X, Zhen Z, Wang X, Guo N (2017). Synergy of a combination of nisin and citric acid against Staphylococcus aureus and Listeria monocytogenes. Food Addit. Contam. Part A.

[40] Field D (2016). In vitro activities of Nisin and Nisin derivatives alone and in combination with antibiotics against staphylococcus biofilms. Front. Microbiol..

[41] Selegard R (2019). Plantaricins markedly enhance the effects of traditional antibiotics against Staphylococcus epidermidis. Future Microbiol..

[42] Hutchings MI, Truman AW, Wilkinson B (2019). Antibiotics: Past, present and future. Curr. Opin. Microbiol..

[43] Renwick MJ, Brogan DM, Mossialos E (2016). A systematic review and critical assessment of incentive strategies for discovery and development of novel antibiotics. J. Antibiot..

[44] Joo HS, Fu CI, Otto M (2016). Bacterial strategies of resistance to antimicrobial peptides. Philos. Trans. R. Soc. Lond. B.

[45] Vaara M (2009). New approaches in peptide antibiotics. Curr. Opin. Pharmacol.

[46] Luong HX, Thanh TT, Tran TH (2020). Antimicrobial peptides: Advances in development of therapeutic applications. Life Sci..

[47] Maldonado A, Ruiz-Barba JL, Jimenez-Diaz R (2003). Purification and genetic characterization of plantaricin NC8, a novel coculture-inducible two-peptide bacteriocin from Lactobacillus plantarum NC8. Appl. Environ. Microbiol..

