# Supplementary material for: Development of novel broad-spectrum antimicrobial lipopeptides derived from plantaricin NC8 β
Source: Sci Rep. 2023 Mar 13;13:4104. doi: 10.1038/s41598-023-31185-8 (PMC10011573; doi:10.1038/s41598-023-31185-8)
Supplement: Supplementary file 1 — Supplementary Information. [file 41598_2023_31185_MOESM1_ESM.docx]

Development of Novel Broad-spectrum Antimicrobial Lipopeptides Derived from Plantaricin NC8 β

Supplementary data

Emanuel Wiman^1^, Elisa Zattarin^2^, Daniel Aili^2^, Torbjörn Bengtsson^1^, Robert Selegård^2,^*, Hazem Khalaf^1,^*

^1^ School of Medical Sciences, Faculty of Medicine and Health, Department of Microbiology, Immunology and Reproductive science, Örebro University, Örebro, Sweden.

^2^ Laboratory of Molecular Materials, Division of Biophysics and Bioengineering, Department of Physics, Chemistry and Biology, Linköping University, SE-581 83 Linköping, Sweden.

*Corresponding authors


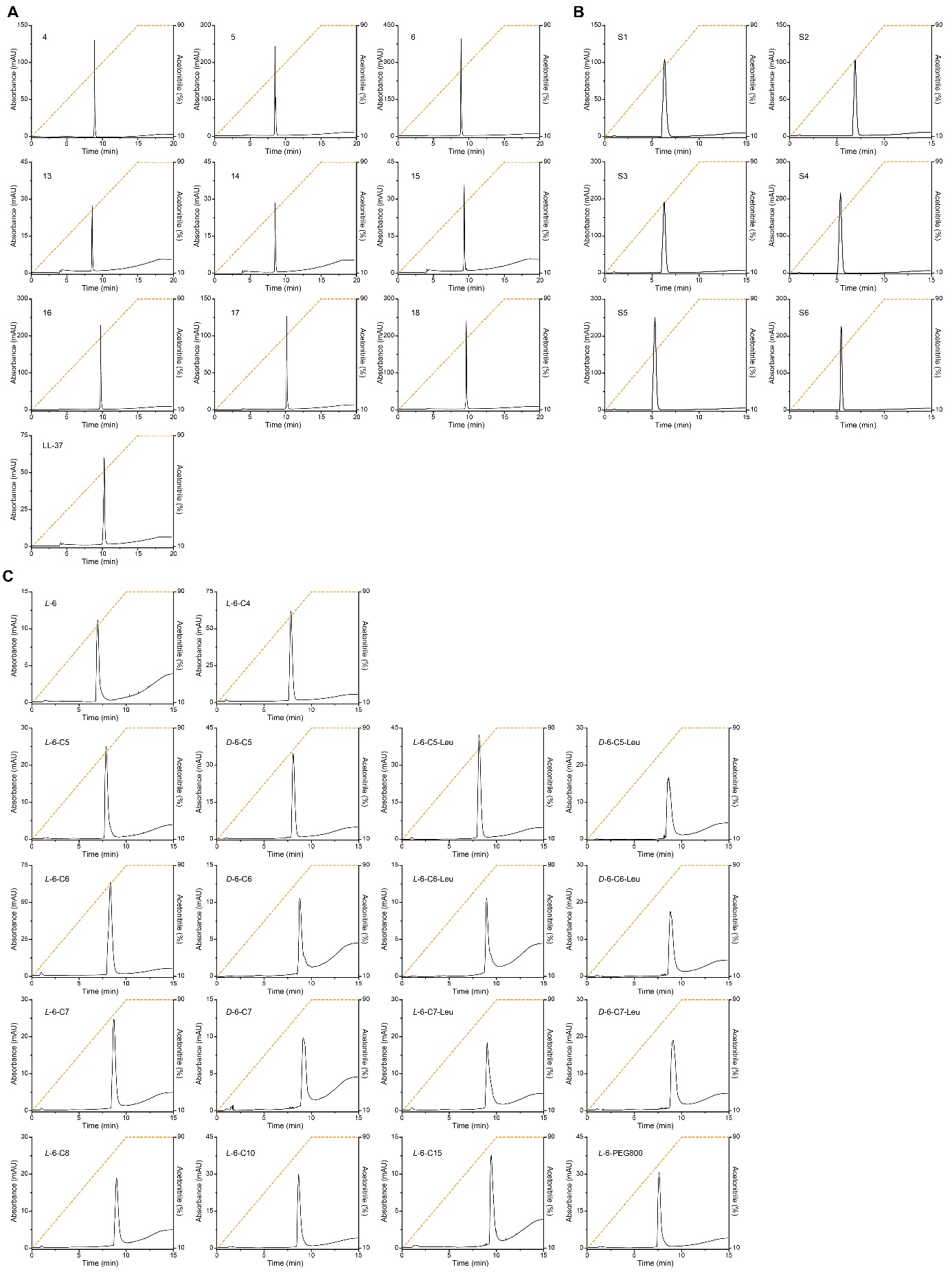


**Figure S1.** **Peptide purity.** HPLC traces of (A and B) synthesized peptides derived from antimicrobial activity prediction shown in table 1, and (C) lipopeptides derived from peptide 6. Orange dotted line indicates acetonitrile gradient (10-90% followed by a linear flow of 90% for 5 min).


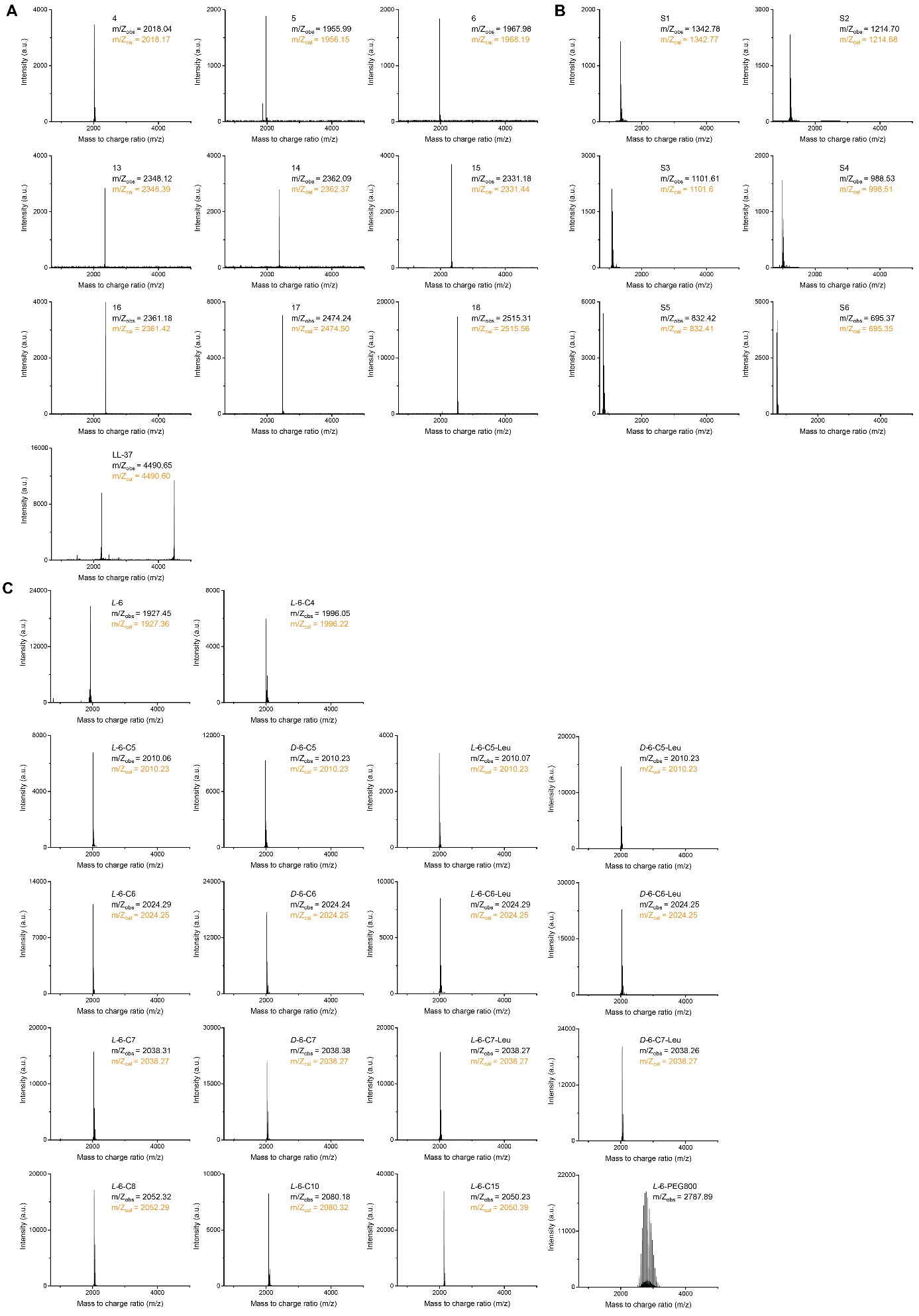


**Figure S2.** **Peptide identity.** MALDI-ToF MS of (A and B) synthesized peptides derived from antimicrobial activity prediction shown in table 1, and (C) lipopeptides derived from peptide 6.

**
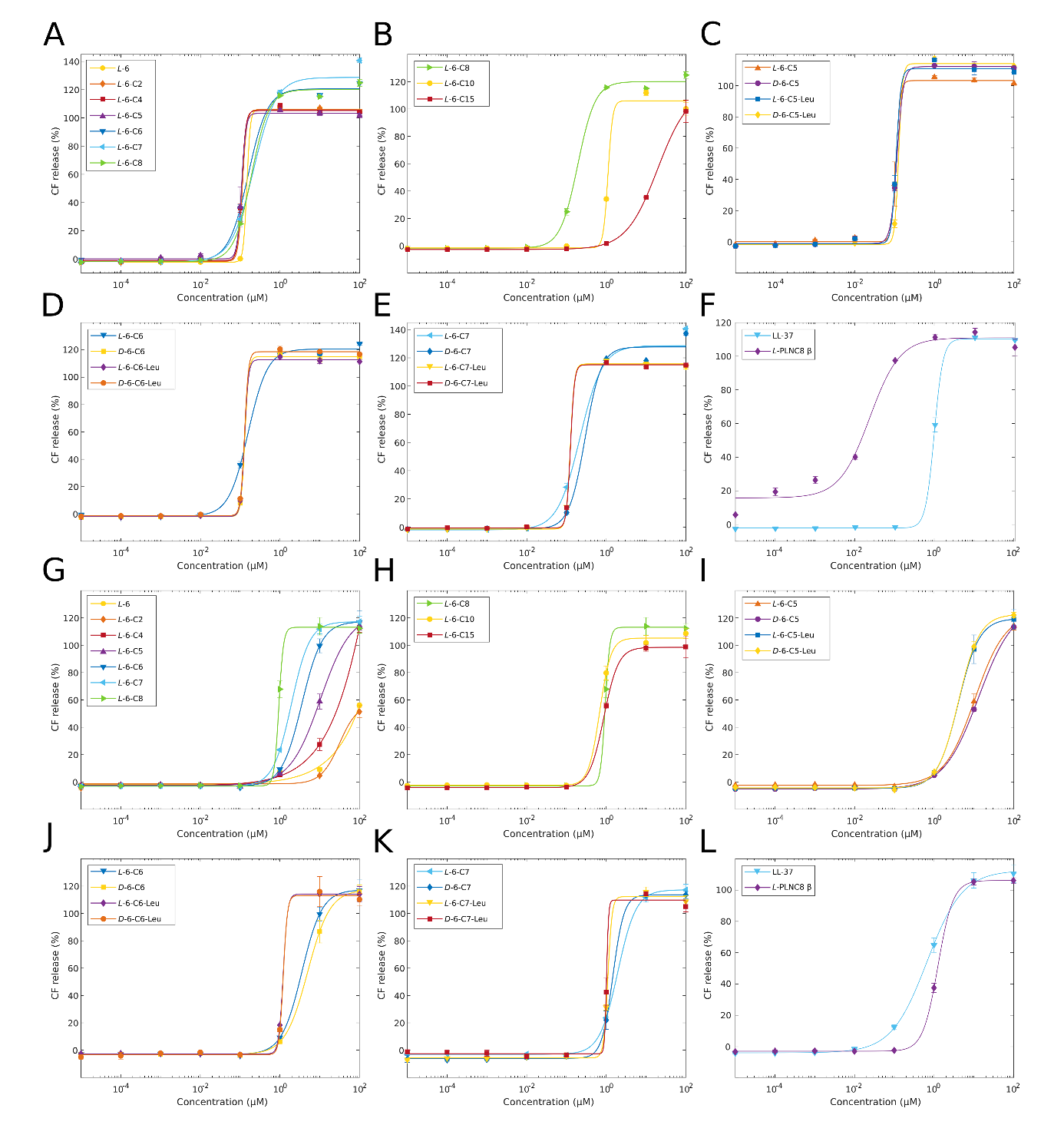
**

**Figure S3.** **Membrane activity**. CF release after 1h incubation of 10^-5^ – 10^2^ µM lipopeptides with 25 µM of **A-F**) bacterial-mimicking liposomes and **G-L**) mammalian-mimicking liposomes in PBS buffer (10 mM, pH 7.4).

**
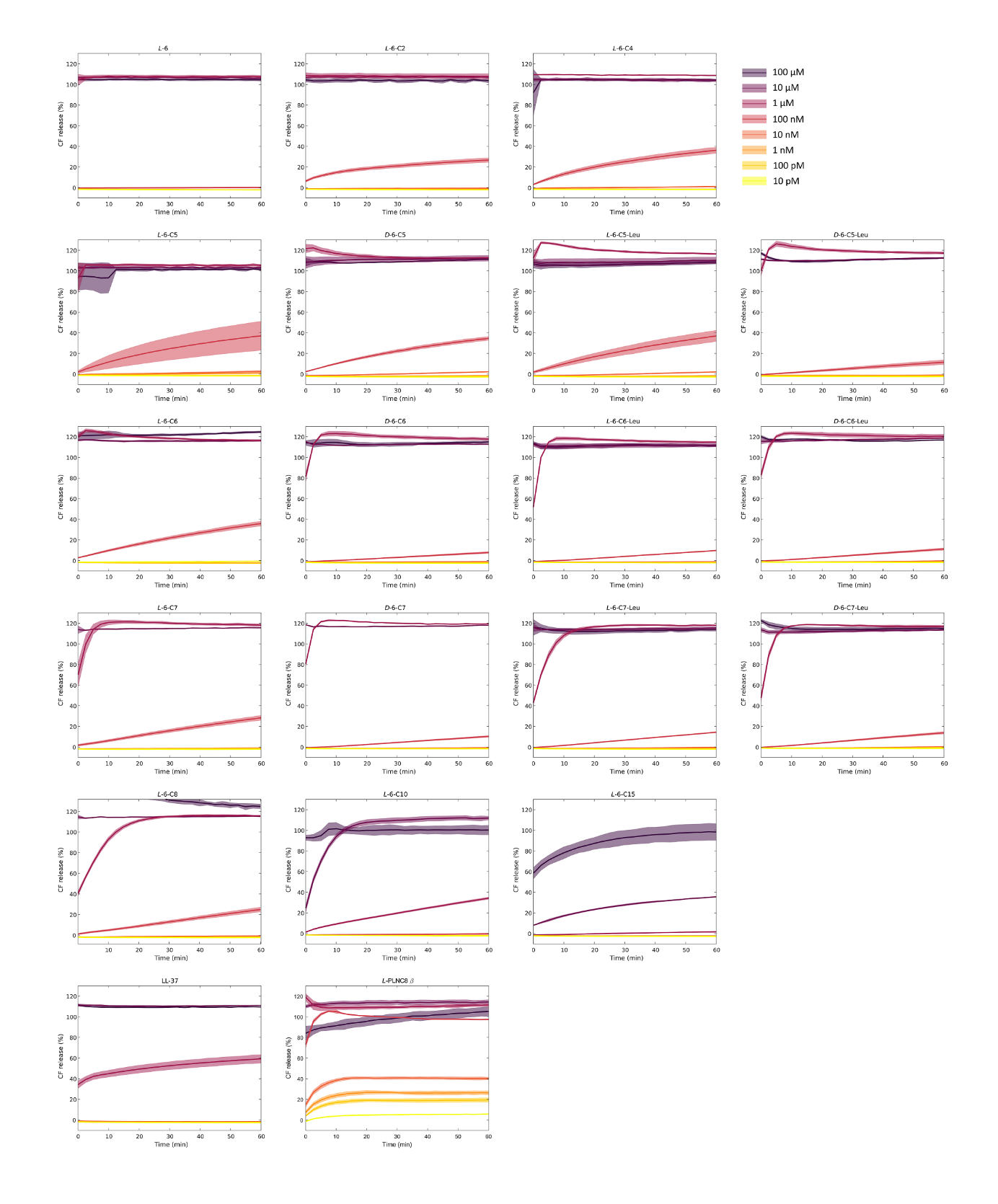
**

**Figure S4.** **Kinetics of membrane activity**. Interaction kinetics between lipopeptides (10^-5^ – 10^2^ µM) and bacterial-mimicking liposomes (25 µM in 10 mM PBS, pH 7.4).


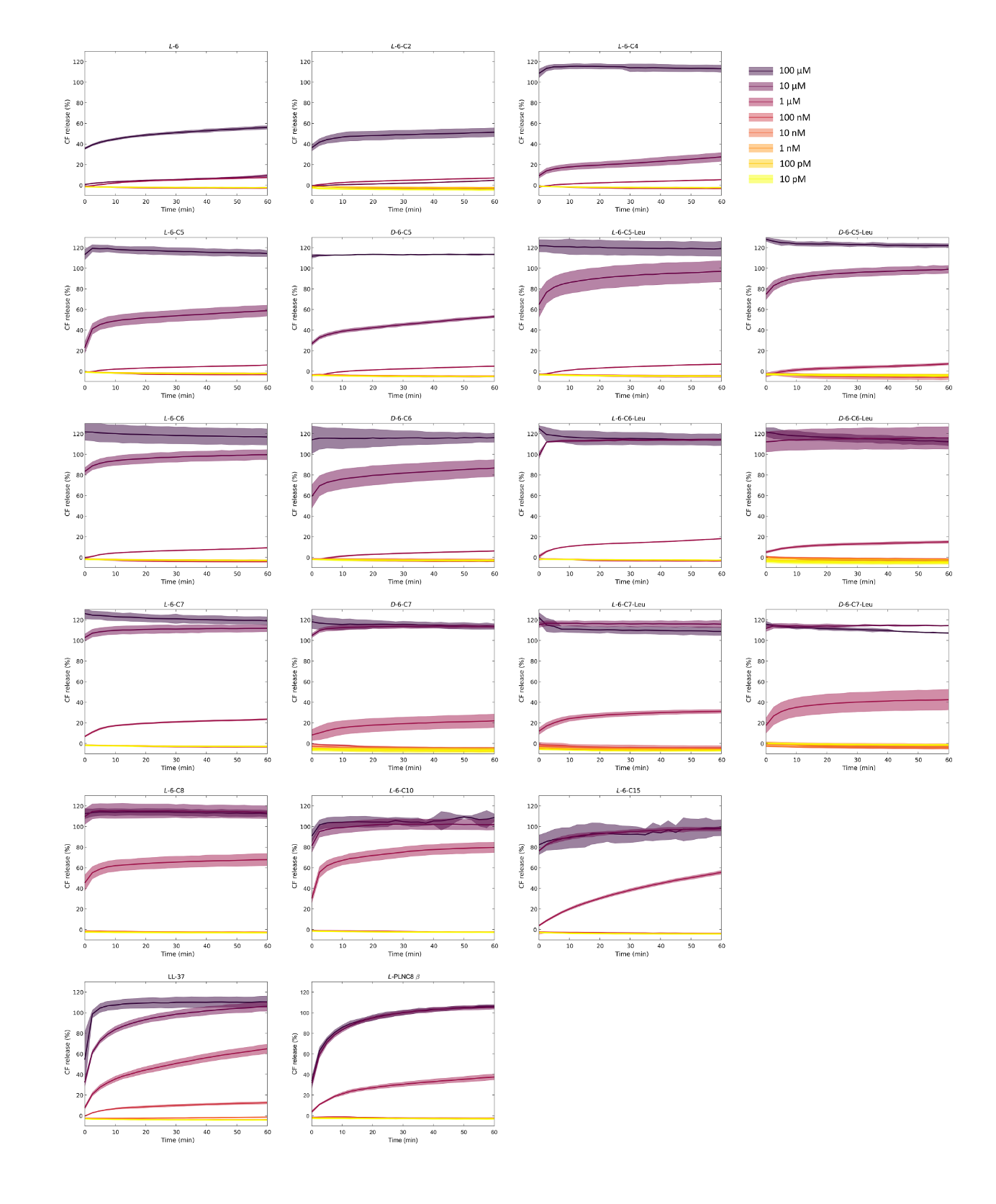


**Figure S5** **Kinetics of membrane activity**. Interaction kinetics between lipopeptides (10^-5^ – 10^2^ µM) and mammalian-mimicking liposomes (25 µM in 10 mM PBS, pH 7.4).

**Table S1.** Resistance patterns and harvest locale for clinical isolates of *S. aureus*, *E. coli* and the ESKAPE pathogens obtained from the Department of Laboratory Medicine at Örebro University Hospital.

| ***S. aureus*** |  | **Origin** | **Iso** | **Fus** | **Kli** | **Gent** | **Rif** | **Trim** | **Cipr** | **Van** | **Dapt** | **Lin** |  |  |  |  |  |
| --- | --- | --- | --- | --- | --- | --- | --- | --- | --- | --- | --- | --- | --- | --- | --- | --- | --- |
| **505018179796** |  | **Blood** | **S** | **S** | **S** | **S** | **S** | **S** | **S** | **S** | **S** | **S** |  |  |  |  |  |
| **505018186054** |  | **Blood** | **S** | **S** | **S** | **S** | **S** | **S** | **S** | **S** | **S** | **S** |  |  |  |  |  |
| **505018193648** |  | **Blood** | **S** | **S** | **S** | **S** | **S** | **S** | **S** | **S** | **S** | **S** |  |  |  |  |  |
| **505018190785** |  | **Blood** | **S** | **S** | **S** | **S** | **S** | **S** | **S** | **S** | **S** | **S** |  |  |  |  |  |
| **505018194921** |  | **Blood** | **S** | **S** | **S** | **S** | **S** | **S** | **S** | **S** | **S** | **S** |  |  |  |  |  |
| **YSAR-21-2843** |  | **Wound** | **S** | **S** | **S** | **S** | **S** | **S** | **S** | **S** | **S** | **S** |  |  |  |  |  |
| **DSAR-21-1211** |  | **Wound** | **S** | **S** | **S** | **S** | **S** | **S** | **S** | **S** | **S** | **S** |  |  |  |  |  |
| **YSAR-21-4907** |  | **Wound** | **S** | **S** | **S** | **S** | **S** | **S** | **S** | **S** | **S** | **S** |  |  |  |  |  |
| **YSAR-21-4909** |  | **Wound** | **S** | **S** | **S** | **S** | **S** | **S** | **S** | **S** | **S** | **S** |  |  |  |  |  |
| **505017845260** | **MRSA** | **Blood** | **R** | **S** | **S** | **S** | **S** | **S** | **S** | **S** | **S** | **S** |  |  |  |  |  |
| **505018733346** | **MRSA** | **Blood** | **R** | **R** | **S** | **S** | **S** | **S** | **S** | **S** | **S** | **S** |  |  |  |  |  |
| **505019733436** | **MRSA** | **Blood** | **R** | **S** | **S** | **S** | **S** | **S** | **I** | **S** | **S** | **S** |  |  |  |  |  |
| **505019741940** | **MRSA** | **Blood** | **R** | **S** | **S** | **S** | **S** | **S** | **I** | **S** | **S** | **S** |  |  |  |  |  |
| **505019945723** | **MRSA** | **Blood** | **R** | **S** | **S** | **S** | **S** | **S** | **I** | **S** | **S** | **S** |  |  |  |  |  |
| **YSAR-21-5398** | **MRSA** | **Wound** | **R** | **S** | **S** | **S** | **S** | **S** | **I** | **S** | **S** | **S** |  |  |  |  |  |
| **YSAR-20-4572** | **MRSA** | **Wound** | **R** | **R** | **R** | **S** | **S** | **S** | **S** | **S** | **S** | **S** |  |  |  |  |  |
| **YSAR-20-5634** | **MRSA** | **Wound** | **R** | **R** | **R** | **R** | **S** | **S** | **R** | **S** | **R** | **S** |  |  |  |  |  |
| **MRSS-20-1637** | **MRSA** | **Wound** | **R** | **R** | **S** | **R** | **S** | **S** | **S** | **S** | **S** | **S** |  |  |  |  |  |
| ***E. coli*** |  |  | **Nit** | **Trpm** | **Cefo** | **Cipr** | **Ceft** | **Pip/Taz** | **Imi** | **Cef** | **Mer** | **Gent** |  |  |  |  |  |
| **1250800204** |  | **UTI** | **S** | **S** | **S** | **S** | **N/A** | **N/A** | **N/A** | **N/A** | **N/A** | **N/A** |  |  |  |  |  |
| **1257800639** |  | **UTI** | **S** | **S** | **S** | **S** | **S** | **S** | **S** | **S** | **S** | **S** |  |  |  |  |  |
| **1257800519** |  | **UTI** | **S** | **S** | **S** | **S** | **N/A** | **N/A** | **N/A** | **N/A** | **N/A** | **N/A** |  |  |  |  |  |
| **1157808982** |  | **UTI** | **S** | **S** | **S** | **S** | **S** | **S** | **S** | **S** | **S** | **S** |  |  |  |  |  |
| **1250800910** |  | **UTI** | **S** | **S** | **S** | **S** | **S** | **S** | **S** | **S** | **S** | **S** |  |  |  |  |  |
| **1257800244** |  | **UTI** | **S** | **S** | **S** | **S** | **S** | **S** | **S** | **S** | **S** | **S** |  |  |  |  |  |
| **1150822152** |  | **UTI** | **S** | **R** | **S** | **S** | **N/A** | **N/A** | **N/A** | **S** | **N/A** | **N/A** |  |  |  |  |  |
| **1257800301** |  | **UTI** | **S** | **S** | **S** | **S** | **S** | **S** | **S** | **S** | **S** | **S** |  |  |  |  |  |
| **1257800601** |  | **UTI** | **S** | **S** | **S** | **S** | **S** | **S** | **S** | **S** | **S** | **S** |  |  |  |  |  |
| **1157809008** |  | **UTI** | **S** | **S** | **S** | **S** | **N/A** | **N/A** | **N/A** | **N/A** | **N/A** | **S** | **Mec** |  |  |  |  |
| **07T-0846** | **ESBL** | **UTI** | **S** | **R** | **R** | **R** | **R** | **R** | **S** | **N/A** | **N/A** | **S** | **R** |  |  |  |  |
| **07T-1105** | **ESBL** | **UTI** | **S** | **R** | **R** | **I** | **R** | **R** | **S** | **N/A** | **S** | **S** | **R** |  |  |  |  |
| **08T-0189** | **ESBL** | **UTI** | **S** | **R** | **R** | **R** | **R** | **R** | **S** | **R** | **S** | **S** | **R** |  |  |  |  |
| **08T-0315** | **ESBL** | **UTI** | **S** | **R** | **R** | **R** | **R** | **R** | **S** | **R** | **S** | **R** | **N/A** |  |  |  |  |
| **08T-0855** | **ESBL** | **UTI** | **S** | **S** | **R** | **I** | **R** | **N/A** | **S** | **R** | **S** | **S** | **S** |  |  |  |  |
| **09B-0005** | **ESBL** | **UTI** | **N/A** | **R** | **R** | **R** | **R** | **N/A** | **S** | **R** | **S** | **S** | **S** |  |  |  |  |
| **07T-1294** | **ESBL** | **UTI** | **S** | **R** | **R** | **R** | **R** | **R** | **S** | **R** | **S** | **S** | **R** |  |  |  |  |
| **07T-0246** | **ESBL** | **UTI** | **S** | **R** | **R** | **R** | **R** | **N/A** | **S** | **N/A** | **N/A** | **R** | **N/A** |  |  |  |  |
|  |  |  | **Nit** | **Trpm** | **Cefo** | **Cipr** | **Ceft** | **Pip/Taz** | **Imi** | **Cef** | **Mer** | **Gent** | **Lin** | **Tge** | **Van** | **Amp** | **Akd** |
| ***E. faecium*** |  | **N/A** | **N/A** | **N/A** | **N/A** | **N/A** | **N/A** | **R** | **R** | **N/A** | **N/A** | **R** | **S** | **S** | **S** | **R** |  |
| ***K. pneumoniae*** |  | **N/A** | **N/A** | **N/A** | **S** | **S** | **S** | **S** | **S** | **S** | **S** | **S** | **N/A** | **N/A** | **N/A** | **N/A** | **S** |
| ***A. baumannii*** |  | **N/A** | **N/A** | **N/A** | **N/A** | **R** | **N/A** | **N/A** | **R** | **N/A** | **R** | **R** | **N/A** | **N/A** | **N/A** | **N/A** | **S** |
| ***P. aeruginosa*** |  | **N/A** | **N/A** | **N/A** | **N/A** | **S** | **S** | **S** | **S** | **N/A** | **S** | **S** | **N/A** | **N/A** | **N/A** | **N/A** | **S** |
| ***E. cloacae-ECC*** |  | **N/A** | **N/A** | **N/A** | **N/A** | **S** | **S** | **S** | **S** |  | **S** | **S** | **N/A** | **N/A** | **N/A** | **N/A** | **S** |
|  |  |  |  |  |  |  |  |  |  |  |  |  |  |  |  |  |  |
| **Key** | |  |  |  |  |  |  |  |  |  |  |  |  |  |  |  |  |
| **Isoxa-Pc** | **Iso** |  |  |  |  |  |  |  |  |  |  |  |  |  |  |  |  |
| **Fusidic acid** | **Fus** |  |  |  |  |  |  |  |  |  |  |  |  |  |  |  |  |
| **Klindamycin** | **Kli** |  |  |  |  |  |  |  |  |  |  |  |  |  |  |  |  |
| **Gentamicin** | **Gent** |  |  |  |  |  |  |  |  |  |  |  |  |  |  |  |  |
| **Rifampicin** | **Rif** |  |  |  |  |  |  |  |  |  |  |  |  |  |  |  |  |
| **TrimSulfa** | **Trim** |  |  |  |  |  |  |  |  |  |  |  |  |  |  |  |  |
| **Ciprofloxacin** | **Cipr** |  |  |  |  |  |  |  |  |  |  |  |  |  |  |  |  |
| **Vancomycin** | **Van** |  |  |  |  |  |  |  |  |  |  |  |  |  |  |  |  |
| **Daptomycin** | **Dapt** |  |  |  |  |  |  |  |  |  |  |  |  |  |  |  |  |
| **Linezolid** | **Lin** |  |  |  |  |  |  |  |  |  |  |  |  |  |  |  |  |
| **Nitrofurantoin** | **Nit** |  |  |  |  |  |  |  |  |  |  |  |  |  |  |  |  |
| **Trimetoprim** | **Trpm** |  |  |  |  |  |  |  |  |  |  |  |  |  |  |  |  |
| **Cefotaxim** | **Cefo** |  |  |  |  |  |  |  |  |  |  |  |  |  |  |  |  |
| **Ciprofloxacin** | **Cipr** |  |  |  |  |  |  |  |  |  |  |  |  |  |  |  |  |
| **Ceftazidim** | **Ceft** |  |  |  |  |  |  |  |  |  |  |  |  |  |  |  |  |
| **Piperacillin/Tazob.** | **Pip/Taz** | |  |  |  |  |  |  |  |  |  |  |  |  |  |  |  |
| **Imipenem** | **Imi** |  |  |  |  |  |  |  |  |  |  |  |  |  |  |  |  |
| **Ceftibuten** | **Cef** |  |  |  |  |  |  |  |  |  |  |  |  |  |  |  |  |
| **Meropenem** | **Mer** |  |  |  |  |  |  |  |  |  |  |  |  |  |  |  |  |
| **Gentamicin** | **Gent** |  |  |  |  |  |  |  |  |  |  |  |  |  |  |  |  |
| **Mecillinam** | **Mec** |  |  |  |  |  |  |  |  |  |  |  |  |  |  |  |  |
| **Amikacin** | **Akd** |  |  |  |  |  |  |  |  |  |  |  |  |  |  |  |  |
| **Tigecyklin** | **Tge** |  |  |  |  |  |  |  |  |  |  |  |  |  |  |  |  |
| **Ampicillin/Amoxicillin** | **Amp** |  |  |  |  |  |  |  |  |  |  |  |  |  |  |  |  |

**Table S2.** Resistance development serial passage assay. MIC- and MBC-values of *S. aureus* and *E. coli* cultured in the presence of sub-mic concentrations (1 µM) of *L*-6-C5 in LB-broth for 30 passages compared to unexposed bacteria (passage 0).

|  | ***L*-6-C5 (µM)** | |
| --- | --- | --- |
|  | **MIC** | **MBC** |
| *S. aureus p0* | **6.3** | **6.3** |
| *E. coli*, p0 | **6.3** | **6.3** |
| *S. aureus p10* | **6.3** | **6.3** |
| *E. coli*, p10 | **6.3** | **6.3** |
| *S. aureus p20* | **6.3** | **6.3** |
| *E. coli*, p20 | **6.3** | **6.3** |
| *S. aureus p30* | **6.3** | **6.3** |
| *E. coli*, p30 | **6.3** | **6.3** |
